# A Targeted Gene Panel That Covers Coding, Non-coding and Short Tandem Repeat Regions Improves the Diagnosis of Patients With Neurodegenerative Diseases

**DOI:** 10.3389/fnins.2019.01324

**Published:** 2019-12-11

**Authors:** Allen Chi-Shing Yu, Aldrin Kay-Yuen Yim, Anne Yin-Yan Chan, Liz Y. P. Yuen, Wing Chi Au, Timothy H. T. Cheng, Xiao Lin, Jing-Woei Li, Larry W. L. Chan, Vincent C. T. Mok, Ting-Fung Chan, Ho Yin Edwin Chan

**Affiliations:** ^1^Codex Genetics Limited, Shatin, Hong Kong; ^2^School of Life Sciences, The Chinese University of Hong Kong, Shatin, China; ^3^Computational and System Biology Program, Washington University School of Medicine, Saint Louis, MO, United States; ^4^Division of Neurology, Department of Medicine and Therapeutics, The Chinese University of Hong Kong, Shatin, China; ^5^Department of Chemical Pathology, The Chinese University of Hong Kong, Shatin, China; ^6^Gerald Choa Neuroscience Centre, The Chinese University of Hong Kong, Shatin, China; ^7^Alice Ho Miu Ling Nethersole Hospital, Tai Po, Hong Kong

**Keywords:** neurodegenerative diseases, undiagnosed diseases, gene panel, short tandem repeat, clinical decision support, high-throughput sequencing

## Abstract

Genetic testing for neurodegenerative diseases (NDs) is highly challenging because of genetic heterogeneity and overlapping manifestations. Targeted-gene panels (TGPs), coupled with next-generation sequencing (NGS), can facilitate the profiling of a large repertoire of ND-related genes. Due to the technical limitations inherent in NGS and TGPs, short tandem repeat (STR) variations are often ignored. However, STR expansions are known to cause such NDs as Huntington’s disease and spinocerebellar ataxias type 3 (SCA3). Here, we studied the clinical utility of a custom-made TGP that targets 199 NDs and 311 ND-associated genes on 118 undiagnosed patients. At least one known or likely pathogenic variation was found in 54 patients; 27 patients demonstrated clinical profiles that matched the variants; and 16 patients whose original diagnosis were refined. A high concordance of variant calling were observed when comparing the results from TGP and whole-exome sequencing of four patients. Our in-house STR detection algorithm has reached a specificity of 0.88 and a sensitivity of 0.82 in our SCA3 cohort. This study also uncovered a trove of novel and recurrent variants that may enrich the repertoire of ND-related genetic markers. We propose that a combined comprehensive TGPs-bioinformatics pipeline can improve the clinical diagnosis of NDs.

## Introduction

Population growth and aging play pivotal roles in dynamic changes in disease patterns. The increasing average age of the world population is accompanied by an epidemiological shift in disease burden from communicable to non-communicable diseases, specifically neurological disorders. Neurodegenerative diseases (NDs) represent a highly heterogeneous group of disorders that are characterized by progressive degeneration of the nervous systems. The major difficulty in diagnosing NDs is the dichotomy between familial (rare) and common (idiopathic/sporadic) cases. Although the underlying genetic profiles in sporadic cases are quite complex, the convoluted clinical symptoms shared among sporadic ND cases often add to the challenge of diagnosis. Previous studies have demonstrated the utility of a microarray-based genotyping approach for the investigation of genetic variations in NDs (Ghani et al., 2015; Nalls et al., 2015; Blauwendraat et al., 2017), but microarray approaches are not well-suited for the detection of insertions and deletions (indels), short tandem repeats (STRs), and novel variants in disease genes. Therefore, it is important to explore more advanced technologies in profiling the genotypes of patients.

The recent advances in next-generation sequencing (NGS) technologies greatly facilitate the identification of mutations that cause various forms of hereditary NDs (Tazir et al., 2014; Tsoi et al., 2014; Yu et al., 2016). Defining the genetic cause of hereditary NDs via NGS platforms avoids excessive diagnostic tests, allows patients to receive the most appropriate clinical care based on the molecular diagnosis, provides the opportunity for genetic counseling of the patients’ families, and allows for a more defined set of patients to be recruited for clinical trials. The three most common clinical NGS diagnostic approaches include whole-genome sequencing (WGS), whole-exome sequencing (WES), and targeted NGS (Klein and Foroud, 2017). Of these three, targeted NGS has become more popular as a diagnostic methodology in many clinical molecular diagnostics settings but is still gaining traction as a routine diagnostic tool in neurological clinics. Compared with WES, custom-designed NGS panels cause less confusion to clinicians. After patients have been clinically diagnosed, a defined set of targeted gene panels can be used to pinpoint the disease-causing mutation. In contrast, WGS and WES produce large numbers of variants with uncertain significance or incidental findings, adding to the challenge of interpreting them in the clinical setting.

Empowered by affordable NGS technologies, reports have accumulated in recent years of cases of successful identification of causative genetic variants underlying a diverse array of human diseases. We have adopted a targeted-gene approach and designed a targeted-gene panel (TGP) focusing on 311 genes that are associated with 199 neurological disorders. Here we report the design of the TGP and the screening results on 118 consenting patients; we have successfully confirmed clinically diagnosed cases and identified potentially causative mutations in unknown ones. Most existing screening methods do not cover the promoter, untranslated regions (UTRs), or intronic regions, which are often linked with neurological diseases (Notebaart et al., 2006; La Spada and Taylor, 2010; Loureiro et al., 2015). Other existing panels that target NDs, such as the ONDRISeq panel (80 genes) (Farhan et al., 2016), the TruSeq Neurodegeneration Panel (118 genes; Illumina Inc) and the panel proposed by Krüger’s group (277 genes) (Krüger et al., 2016), have less genes covered and do not support the analysis of STR. We propose that the extended panel and the bioinformatics analysis pipeline can support the clinical diagnosis of NDs by delineating overlapping observations, enabling clinicians to reduce the time to diagnosis by assessing a wide range of NDs in one test.

## Materials and Methods

### Patient Samples

One hundred eighteen patients were recruited from the adult neurological disease clinic in the Prince of Wales Hospital. According to assessments by at least one neurology specialist, these patients showed various degrees of neurodegeneration, yet definitive diagnosis was not possible despite having the results of standard imaging, biochemical, and target-gene tests. This study was approved by the Joint Chinese University of Hong Kong–New Territories East Cluster Clinical Research Ethics Committee (ref. nos. CRE-2012.361). Informed consent was obtained from all subjects in this study.

### Design of a Targeted Enrichment Panel

We curated a catalog of 199 neurodegenerative disorders and 311 associated genes (Supplementary Table S1) from the Orphanet (INSERM, 1997) and OMIM databases (Hamosh et al., 2005). We emphasized genes that were implicated in ataxias, spastic paraplegia (SPG), Charcot-Marie-Tooth (CMT) disease, amyotrophic lateral sclerosis, and other rare Mendelian NDs. Genes that are linked to more common NDs, such as Alzheimer’s disease (AD) and dementia, were included to aid in delineating the observed symptoms. The TGP covers a total length of 2,453,403 base pairs (bps). The target capture probes for the 6,806 exons, splice regions, UTRs, promoters, and selected introns that are known to be involved in neurological repeat expansions were designed using SeqCap NimbleDesign (Roche, Switzerland).

### Library Preparation and Sequencing

Genomic DNA molecules of at least 1 μg were isolated from a subject’s blood using a QIAamp DNA Blood Mini Kit (QIAGEN Inc). The quantity and quality of the extracted DNA were assessed by NanoDrop 2000 (Thermo Fisher Scientific, United States) and Qubit fluorometer (Thermo Fisher Scientific, United States).

The target-capture method was adapted from Roche SeqCap EZ Library SR User’s guide Version 5.1. To obtain a larger dynamic range of DNA STR variation, the insert size of paired sequencing reads was increased from the default 250 bps to a median of 350 bps. This required tuning the Covaris ultrasonoscope (Covaris, United States) to output fragments of 330–370 bps, followed by reducing the PEG/NaCl SPRI solution ratio to 0.5 volume during the double size selection step with AMPure beads (Beckman Coulter, United States). Each targeted region of the human genome was sequenced at a depth of about 200 times using the Illumina NextSeq 500 paired-end 2 × 150-bp high-throughput DNA sequencing platform.

The whole-exome target capture was performed according to the recommendations of the Roche SeqCap EZ MedExome Kit. Sequencing of the whole-exome library was performed using the Illumina MiSeq paired-end 2 × 75-bp high-throughput DNA sequencing platform.

### Sequence Alignment and Variant Calling

Raw reads were first processed using quality-check programs, such as Trimmomatic, to perform quality trimming and remove adapter contaminants. The short-read sequences were aligned to the human genome (version GRCh38) using BWA (version 0.7.12) (Li and Durbin, 2009), followed by PCR duplicates marking, local realignment around indels, and base quality score recalibration using Picard (version 1.141) and Genome Analysis Toolkit (GATK:version 3.7) (McKenna et al., 2010).

Single-nucleotide polymorphisms (SNPs) and small indels were called using methods that we described previously (Tsoi et al., 2014; Yu et al., 2016), albeit with updated programs and databases. In brief, calling of SNPs and small indels was performed using batch genotype calling of all subjects with GATK v3.7 HaplotypeCaller, which outputs variant sites that can be observed in at least one subject. The variants were filtered according to the following parameters: (1) GATK variant quality filter (PASS); (2) Variants within targeted regions; (3) Allele frequency in population genetics databases <0.01, except those with previously reported pathogenicity in ClinVar (1000 Genome phase 3, GnomAD Exome, NHLBI Exome Sequencing Project 6500); (4) Copy number variations (CNVs) were called using Seq2C and CNVkit (Talevich et al., 2016). In brief, both methods normalize the read depths of the targeted regions in a population of samples and call CNVs in regions that show significant deviations in read depths. CNVkit also adjusts for some common biases, such as repetitive sequences and GC content, and uses off-target reads to help detect CNVs.

### Short Tandem Repeat (STR) Variation Calling

Variations of STRs were first assessed using exSTRa (Tankard et al., 2018). In brief, the exSTRa method assumes that most of the individuals in a tested cohort (>85%) have a normal range of STR lengths. The distribution of STR lengths is further simulated using an empirical method. The statistical significance of the observed STR length of an individual was assessed using an average of multiple *t*-statistics.

The results from exSTRa were far from satisfactory when applied to our sequencing data. We developed an in-house method for the detection of STR variations in the SCA3 locus. We used 11 patients who were screened positive for SCA3 and 36 negative controls for the analysis. The method begins with the calculation of a read count matrix *M* using HTSeq (–non-unique all), where rows correspond to STR loci and columns correspond to samples. Next, the matrix *M* is normalized by the library size of each sample using the trimmed mean of M (TMM) method (Robinson and Oshlack, 2010). Using the R fitdistrplus package (version 1.0), we fit the normalized read counts from the control group to five parametric distributions (normal, uniform, exponential, logistic, beta, lognormal, and gamma). The Cullen and Frey graph (see Supplementary Figure S2) revealed that the normal distribution has the best fit. Maximum likelihood estimation was used to model the parameters of the normal distribution of normalized read counts, where the log-likelihood function is:

$$\ln L\,\left(\mu ,\sigma^{2}\right)=-\frac{n}{2}\,\ln\left(2\,\pi \right)-\frac{n}{2}\,\ln\left(\sigma^{2}\right)-\frac{1}{2\,\sigma^{2}}\,\sum_{i=1}^{n}{\left(x_{i}-\mu \right)}^{2}$$

Moreover, the maximum likelihood estimation of the mean μ and variance σ^2^ are defined as:

$$\hat{\mu }=\frac{\sum_{i=1}^{n}x_{i}}{n}$$

$${\hat{\sigma }}^{2}=\frac{\sum_{i=1}^{n}{\left(x_{i}-\hat{\mu }\right)}^{2}}{n}$$

We used a Z-score-based method to determine if the patients have SCA3.

$$z_{i}=\frac{x_{i}-\hat{\mu }}{\sigma }$$

The Z-score threshold (–0.91) was determined from the turning point of the Receiver Operating Characteristic (ROC) curve, where the sensitivity and specificity are best balanced. A negative *Z*-score below the threshold of –0.91 represents an expanded SCA3 locus.

### Variant Interpretation

Variant annotation was performed using Variant Effect Predictor and dbNSFP (Liu et al., 2013), which included features such as population allele frequencies (dbSNP 150, 1000 Genome phase 3, GnomAD Exome, NHLBI Exome Sequencing Project 6500), sequence conservation (GERP++, PhyloP, SiPhy), and functional impact predictions (including, but not limited to, fathmm-MKL, Sift, PROVEAN, MutationAssessor, MutationTaster, MetaSVM, and MetaLR).

Our scheme of variant classification closely adheres to the recommendation by the American College of Medical Genetics (ACMG) (Richards et al., 2015) and the industry standard among clinical genetics testing laboratories. This scheme represents a framework for the interpretation of variants.

The classification scheme applies to variants in genes associated with diseases. Each variant was individually assessed in the context of the variant, gene, associated disease, and patient phenotype.

Sequence variants are classified in one of four categories (pathogenic, likely pathogenic, benign, and likely benign). If the variant cannot be classified into one of the four categories above, it is classified as of uncertain significance. Benign and likely benign variants are not reported in the report. Pathogenic and likely pathogenic variants discovered in this study were confirmed by Sanger sequencing.

## Results

### Study Design

**FIGURE 1:**
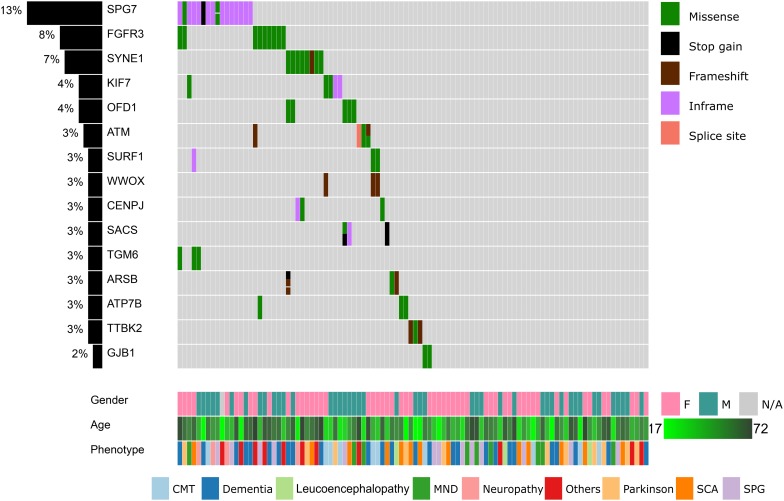
Summary of the top 15 variants, clinical symptoms, and demographic profiles of the recruited patients. Columns represent patients and rows represent genes. The percentage of patients in our cohort having at least one missense, stop gain, frameshift indels, inframe indels, or splice site variation in each gene is shown on the left. Variants, clinical symptoms and demographic profiles are color-coded according to the legends within the figure.

Altogether, 118 patients with various degrees of neurodegenerative features, such as ataxias, neuropathy, and unsteady gait, were recruited from the adult neurological disease clinic in the Prince of Wales Hospital (Figure 1). Each patient was reviewed by at least one specialist in neurology, but definitive molecular diagnosis was not possible even though multiple neuroimaging investigations, biopsy, biochemical tests, and targeted genetic screening had been performed previously. The cohort demonstrated diverse neurological symptoms: 14 patients were referred with SCA, 13 patients were referred with Parkinson’s disease (PD), 12 patients were classified as having AD, 12 patients were classified as having SPG, and 11 patients were pre-diagnosed with CMT disease. The remaining patients demonstrated symptoms such as dementia, motor neuron disease, and muscle atrophy. The full list of patients is available in Supplementary Table S2.

We designed a TGP to cover most genomic regions that have been implicated in NDs, which helps to provide additional molecular genetics information for the refinement of diagnosis. The focused scope of our TGP allows accurate interrogation of genomic variants through deep sequencing and alleviates the uncertainty of diagnosis by including genes with proven clinical value while maintaining a relatively low cost. The TGP covers 6,806 exons, splice regions, UTRs, promoters, and selected introns that have been implicated in ND. Three hundred eleven genes are associated with 199 neurodegenerative disorders according to the Orphanet (INSERM, 1997) and OMIM databases (Hamosh et al., 2005). The full list of NDs covered by the TGP is provided in Supplementary Table S1.

### The Landscape of the Identified Variants

All 118 samples were sequenced using an Illumina NextSeq500 system. The average depth of sequencing was 407.5 ± 36.0, and 99.2% of targeted bases were covered by at least one read (Supplementary Figure S1). The average reads mapping rate was 99.7% ± 0.1%. We identified 16,693 small variants, including 13,235 SNPs and 3,458 indels. When compared to dbSNP release 150, 14,118 variants (84.6%) were previously recorded. These variants can be further classified into 24 nonsense, 66 frameshift indels, 138 inframe indels, 1,671 missense, 34 splice-site, 3 starts lost, 1 stop lost, 1,535 synonymous, and 13,221 non-coding variants.

In our cohort of patients, 54 out of 118 (45.8%) cases demonstrated at least one pathogenic or likely pathogenic variation according to the ClinVar database (version 20190311). These cases shared 44 unique pathogenic/likely pathogenic variants. Upon review by the neurology specialists, 27 cases showed clinical relevance to the observed symptoms. These pathogenic variants were also confirmed by Sanger sequencing (Table 1). The high prevalence of pathogenic/likely pathogenic variants in our ND cohort demonstrated the utility of panel-based high-throughput sequencing analysis, which excels in terms of the breadth of disease coverage when compared to single-gene tests.

**TABLE 1:** List of pathogenic variants and related clinical findings.

	**Patient ID**	**Age of onset (range)**	**Phenotype**	**Gene**	**Variant**	**Reported**	**Relevant specialist investigations**	**MRI performed**	**Final diagnosis**
1	0fd42	36–40	CMT	GJB1	NM_000166.6:c.118G > T	Likely Pathogenic	ncs showed demyelination	no	CMTX1
2	7f225	26–30	CMT	GJB1	NM_000166.5:c.-103C > T	Pathogenic	ncs showed demyelination	no	CMTX1
3	5316c	6–10	CMT	GJB1	NM_000166.5:c.-103C > T	Pathogenic	ncs showed demyelination	no	CMTX1
4	59e19	41–45	CMT	GJB1	NM_000166.5:c.-103C > T	Pathogenic	ncs showed demyelination	no	CMTX1
5	67067	31–35	CMT	GJB1	NM_000166.5:c.-103C > T	Pathogenic	ncs showed demyelination	no	CMTX1
6	44c80	11–15	CMT	GJB1	NM_000166.5:c.-103C > T	Pathogenic	ncs of LL showed unelicited result	no	CMTX1
7	b17ef	36–40	CMT	GJB1	NM_000166.5:c.-103C > T	Pathogenic	ncs showed demyelination	MRI brain SVD	CMTX1
8	c8376	36–40	AD PD	PSEN1 LRRK2	NM_000021.4:c.781G > A NM_198578.3:c.4883G > C	Likely Pathogenic	amyloid PET scan positive	MRI brain SVD	EOAD
9	1da51	46–50	SPG	SPAST	NM_014946.3:c.1507C > T	Pathogenic	no	MRI brain thinning corpus callosum	SPG4
10	785f3	61–65	ALS	TARDBP	NM_007375.3:c.892G > A	Likely Pathogenic	EMG showed neurogenic changes	MRI spine normal	ALS10
11	9400f	6–10	SPG SCA	SACS	NM_014363.5:c.[7504C > T;8132C > T]	Pathogenic	ncs showed demyelination	MRI brain cerebellar atrophy	ARSACS
12	3d914	31–35	SCA	SYNE1	NM_182961.3:c.[20263C > T;8889delT]	Pathogenic	no	MRI brain cerebellar atrophy	SCAR8
13	e3d6c	46–50	SCA	TTBK2	NM_173500.3:c.1306 _1307delGA	Pathogenic	no	MRI brain cerebellar atrophy	SCA11
14	e7f6c	16–20	SCA	TTBK2	NM_173500.3:c.1329dupA	Pathogenic	no	MRI brain cerebellar atrophy	SCA11
15	4e074	UNKNOWN	SCA	PRKCG	NM_002739.4:c.301C > T	Pathogenic	CT brain showed cerebellar atrophy	no	SCA14
16	b1556	41–45	ALS	TARDBP	NM_007375.3:c.892G > A	Pathogenic	no	MRI spine normal	ALS10
17	f5ca3	41–45	SPG	PSEN1	NM_000021.3:c.811C > G	Pathogenic	no	no	AD type 3, with spastic paraparesis
18	76a50	36–40	SCA	LRRK2 POLG	NM_198578.3:c.4883G > C NM_001126131.1:c.2890C > T	Pathogenic	no	no	SCA
19	d59ec	61–65	MND	LRRK2 GDF6	NM_198578.3:c.4883G > C NM_001001557.3: c.1271A > G	Pathogenic	no	no	Parkinson disease 8, Klippel-Feil syndrome 1
20	73475	51–55	Leucoence phalopathy	LRRK2	NM_198578.3:c.7153G > A	Pathogenic	no	no	Parkinson disease 8
21	031b4	56–60	AD	LRRK2	NM_198578.3:c.4883G > C	Pathogenic	no	no	Parkinson disease 8
22	3e1e9	61–65	FTD Progressive Supranuclear Palsy	LRRK2	NM_198578.3:c.4883G > C	Pathogenic	no	no	Parkinson disease 8
23	108c9	UNKNOWN	UNKNOWN	LRRK2	NM_198578.3:c.4883G > C	Pathogenic	no	no	Parkinson disease 8
24	69f59	36–40	PD	LRRK2	NM_198578.3:c.4883G > C	Pathogenic	no	no	Parkinson disease 8
25	482d9	66–70	Mitochondrial disease	PQBP1	NM_001032383.1:c.461_462del	Pathogenic	no	no	Renpenning syndrome 1
26	ef2d1	61–65	SCA	TGM6	NM_198994.2:c.1550T > G	Pathogenic	no	MRI brain cerebellar atrophy	SCA35
27	7688b	66–70	AD	APOE	ApoE-ε4/ε4	Pathogenic	no	MRI brain mild atrophy	LOAD

We proceeded to remove common variants (minor allele frequency <1% in all populations), synonymous variants, non-coding variants, known sequencing artifacts, known non-pathogenic variants, and missense variants that are unanimously predicted to be benign. After these filters were applied, 257 coding variants remained. The top 15 mutated genes are summarized in Figure 1.

Fourteen genes were mutated in more than two patients (Figure 1). The *SPG7* gene, which is involved in SPG 7, was mutated in 16 patients (13%). Most of these patients (13 out of 16) harbored the novel p.Leu8del (NM_003119.3:c.21_23del) variant in the N-terminal signal peptide region. In addition, nine patients (8%) had a p.Asp668Tyr (NM_000142.4:c.2002G > T) variant in the protein kinase domain of FGFR3. In *SYNE1*, four variants (p.Gln2986AsnfsTer13, p.Gln6494Arg, p.Arg5551Leu, p.Ala3030Val) were located in seven patients. *KIF7*, *OFD1*, and *ATM* are also mutated in >3% of our cohort.

We searched further for other recurrent variants that can be found in patients with similar symptoms. The search returned three novel variants. In the first case, we found three heterozygous carriers (ID: 37834, ID: 8c1f1, ID: ad573) of WWOX p.Glu66GlyfsTer3 (NM_016373.4:c.196dup). These three patients had been previously diagnosed with CMT disease. Next, PEX7 p.Gly41Arg heterozygous variant (NM_000288.4:c.121G > C) was found in two unrelated patients (ID: 67067 and ID: a68b4). These two patients demonstrated neuropathy and senile dementia, respectively. Finally, in two unrelated patients (ID: 8c1f1 and ID: 37834) who demonstrated CMT symptoms, we found a novel SURF1 heterozygous p.Gly257Arg (NM_003172.4:c.769G > A) variant. The predicted pathogenicity values of the variants were high according to a majority of predictors. Further experiments were planned to elucidate the functional role of these mutated genes in NDs.

### Variants Are Highly Concordant With Results From WES

To assess the accuracy and reproducibility of the TGP, we chose four samples (ID: 76a50, ID: 4b227, ID: ef2d1, and ID: 79026) that do not belong to the same family to perform WES and to compare that with the TGP results. A total of 1,998 common variants were identified, and within them, 1,895 variants were single-nucleotide variants (SNVs) and 103 were indels. Out of 1,895 SNVs within the four samples shared between the WES and TGP, 1,894 SNVs were identical, leading to a concordance rate of 99.94%. For indels, 96 out of 103 variants (93.2%) had the same indels.

### Changes in Sequencing Depth Inform STR Variations

The detection of STR variations from short reads has been extremely challenging. Early methods (Gymrek et al., 2012; Cao et al., 2015) focused on short STR loci that are within the read length of Illumina’s sequencing platforms. More recently, several methods have become available to detect STR variations that are beyond the length of sequencing reads. Among these methods, exSTRa (Tankard et al., 2018) is the only one that supports target capture–based data, such as WES.

Because our TGP includes capture probes that target neurological repeat loci, as an extended goal, we set out to investigate the possibility of detecting neurological repeat expansions through our platform. To this end, we recruited 11 additional patients who were screened positive for SCA3. The results of these positive cases were compared to 36 negative controls in the original cohort to assess the accuracy. The controls were screened negative for CAG or CTG expansions, including DRPLA, DM1, DM2, SCA1, SCA2, SCA3, SCA6, SCA7, SCA8, and SCA12.

Marked STR expansions were detected in 7 out of 11 SCA3 patients (Table 2). However, the predicted expansion locus was correct in only one of the patients (ID: 3038b). The low specificity of the predictions could be attributed to the difficulty in mapping repetitive sequences. In fact, all of the wrong predictions belong to the class of CAG expansions. Given that SCA3 involves expansion of CAG triplets, in other words, exSTRa correctly predicted CAG expansions in about 64% of cases.

**TABLE 2:** STR expansion predictions on patients with SCA3.

**Patient ID**	**Original**	**exSTRa**	**exSTRa**	**Inhouse**	**Inhouse**
	**diagnosis**	**prediction**	***P*-value**	**prediction**	***Z*-score**
2abac	SCA3	SCA2	1.00E-04	SCA3	−2.00
3038b	SCA3	SCA3	3.00E-04	SCA3	−2.59
A001	SCA3	SCA6,HD	1.00E-04	NA	−0.86
A032	SCA3	NA	NA	SCA3	−2.55
A039	SCA3	NA	NA	SCA3	−1.28
A097	SCA3	NA	NA	SCA3	−1.88
A115	SCA3	SCA2,SCA17	1.00E-04	SCA3	−3.50
A140	SCA3	NA	NA	NA	−0.45
B042	SCA3	SCA2,SCA17	2.00E-04	SCA3	−0.92
B180	SCA3	SCA8	1.00E-04	SCA3	−2.47
P126	SCA3	SCA17	1.00E-04	SCA3	−2.31

**FIGURE 2:**
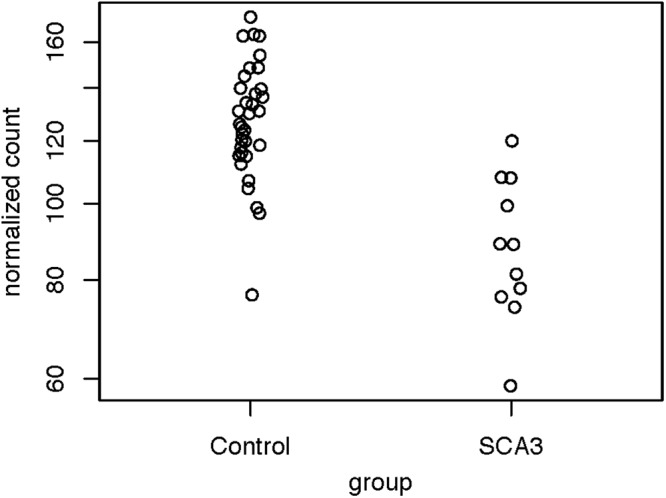
Distribution of normalized read counts of the SCA3 loci. The read counts of the controls and the patients with SCA3 were compared using the Wald test (*P* = 1.47e-05, FDR = 4.42e-05).

Because we were dissatisfied with the accuracy of exSTRa, we attempted to build our method. CAG expansions greatly increase the CG content of the STR loci, causing bias in both the target-capture and sequencing stages (Benjamini and Speed, 2012; Ekblom et al., 2014). Moreover, the CAG expansions would lower sequence mappability because of mismatches with the reference sequence. These factors could manifest in a drop of the depth of coverage. Indeed, by looking at the normalized read count between the SCA3 and the control group (Figure 2), we observed a significant drop in the normalized read count (Wald test: *P* = 1.47e-05, FDR = 4.42e-05).

To check if a sample contained significant SCA3 expansion, we developed a Z-score-based method to screen the patients. First, we fitted the normalized read counts from the control group to a parametric distribution. The Cullen and Frey graph (Supplementary Figure S3) revealed that the distribution of the normalized read counts closely resemble a normal distribution. Therefore, we applied the maximum likelihood estimation to model the read counts as a normal distribution. The Q-Q plot (Supplementary Figure S4) showed that all data points fall closely along the diagonal, which signifies a good fit.

**FIGURE 3:**
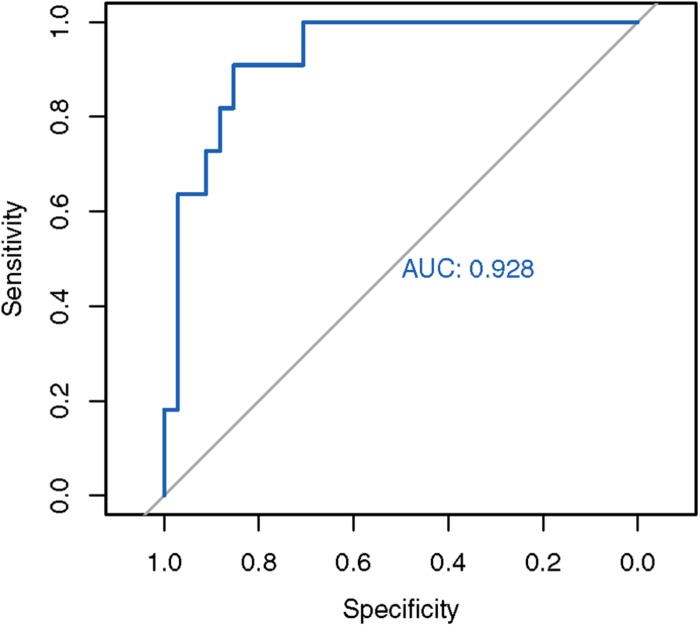
ROC analysis of the in-house SCA3 detection algorithm. The in-house *Z*-score method achieved an AUC of 0.928.

Next, we used the mean and standard deviations from the fitted distribution to calculate a *Z*-score for each known SCA3 sample. A highly negative *Z*-score represents a highly expanded SCA3 locus. The method achieved an AUC of 0.928 from analyzing the ROC curve (Figure 3). We chose the turning point of the ROC curve as the Z-score threshold (–0.91) for SCA3 classification. In other words, any sample with a score below –0.91 would be classified as SCA3. We achieved a specificity of 0.88 and a sensitivity of 0.82 in our cohort (Table 2).

## Discussion

This study highlights the utility of panel-based screening for undiagnosed patients with neurological symptoms. We identified 49 (41.5%) patients who carry at least one pathogenic variation or likely pathogenic variation. Upon further clinical follow-up, 27 (22.9%) of these patients demonstrated phenotypes that matched with the variants (Table 1). Of note, about one third of our patients presented with complex phenotypes, such as AD, frontotemporal dementia (FTD), and PD. As complex NDs are not caused by genetic factors alone, the integration of non-genetic factors in the clinical decision process could improve the diagnostic yield.

### Refinement of Diagnosis

Due to genetic heterogeneity and overlapping symptoms, the diagnosis of NDs is highly challenging. For example, there are over 40 subtypes of spinocerebellar ataxia (SCA). While progressive ataxia is a common clinical observation of SCA, each subtype has its own distinctive genetic marker (Soong and Morrison, 2018). Patients with Huntington’s disease with a clinical presentation of ataxia are often mistaken for SCA (Dong et al., 2013). Due to slow progression of many NDs, definitive clinical features may not appear at onset (Armstrong et al., 2005). These factors highlight the need of a genetic screening method that can cover a broad range of genes related to NDs for the refinement of diagnosis.

Because our TGP allowed the interrogation of variants in 311 genes in one test, it facilitated the refinement of the preliminary diagnosis of 16 patients (Supplementary Table S1). Here, we highlight a few cases that involved the discovery of novel variants. In a female patient (ID: c8376) who was suspected of having FTD or PD, we found a novel heterozygous PSEN1 p.Val261Ile variant (NM_000021.4:c.781G > A). This novel variant is not recorded in population genetics databases, such as 1000 Genome database Phase III (1000G) and Genome Aggregation Database (gnomAD). It is located in a highly conserved position within the presenilin domain. The predicted functional impact of the variant is high according to multiple *in silico* predictors. Variants in *PSEN1* cause up to 70% of early-onset AD (Larner and Doran, 2006). Val261Phe and Val261Leu were discovered in six families, which perfectly segregated with AD (Farlow et al., 2000; Rogaeva et al., 2001; Miravalle et al., 2002; Jiménez Caballero et al., 2008; Gómez-Tortosa et al., 2010; Cruts et al., 2012). A pathogenic variant (Ala260Val) at the immediate upstream amino acid position was also found in a Japanese early-onset AD family (Ikeda et al., 1996). Consistent with these case reports, the age of onset of our patient was 40. A PET scan revealed amyloid-β plaque accumulation in the patient. An MRI scan of the brain showed features that are consistent with small-vessel disease. Given these pieces of evidence, this novel variant was classified as a likely pathogenic variant (PM1, PM2, PM5, PP3, PP4) according to the ACMG guidelines (Richards et al., 2015). The diagnosis of the female patient (ID: c8376) was thus refined to early-onset AD.

In a male patient (ID: 0fd42) who was suspected of having CMT disease, we were able to refine the subtype. A hemizygous GJB1 (NM_000166.6:c.118G > T) Ala40Ser variant was discovered in the patient. The novel Ala40Ser variant was not found in the 1000 Genome database Phase III (1000G) or the Genome Aggregation Database (gnomAD). The variant was located at a highly conserved position in the connexin domain. The predicted pathogenicity is high according to multiple *in silico* predictors. Pathogenic variants were also reported in the amino acids immediately upstream (p.Ala39Val; ClinVar ID: 188136) and downstream (p.Glu41Asp; ClinVar ID: 21079). Functional studies of a different substitution at the same amino acid position (p.Ala40Val) showed that the mutant protein is located in the Golgi apparatus without contacting the cell membrane, and might interfere with the formation of gap junctions as a result of trafficking abnormalities (Yum et al., 2002). Based on multiple lines of evidence, this novel variant was classified as a likely pathogenic variant (PM1, PM2, PM5, PP3, PP4) according to the ACMG guidelines (Richards et al., 2015). Therefore, the diagnosis of the patient was refined to CMT neuropathy X-linked.

In one patient (ID: 3d914), we observed novel variants that helped to refine the preliminary diagnosis of cerebellar degeneration. In 3d914, we observed a novel heterozygous p.Gln2964AsnfsTer13 variant (NM_182961.3:c.8889delT) that pairs with a known p.Arg6755Ter likely pathogenic variant (ClinVar ID: 424802) in SYNE1. This novel variant was not recorded in population genetics databases. Previous research has demonstrated that compound heterozygous variants in *SYNE1* are linked to SCA autosomal recessive 8 (SCAR8) (Lee et al., 2014). Indeed, this patient demonstrated classic phenotypes of SCAR8, including cerebellar atrophy detected by MRI. Here, we showed that this novel compound heterozygous variant pair can be classified as pathogenic (PVS1, PM2, PM3, PP4) according to the ACMG guidelines (Richards et al., 2015).

Similarly, in another patient (ID: 9400f), who displayed ataxia and spastic gait, we observed a novel SACS p.Ser2711Leu variant (NM_014363.5:c.8132C > T) in compound heterozygous configuration with a known pathogenic variant (p.Arg2502^∗^; ClinVar ID: 5513). The variant pair can be classified as likely pathogenic (PM2, PM3, PP3, PP4) according to the ACMG guidelines (Richards et al., 2015). The patient’s nerve conduction study showed demyelination, and the MRI scan demonstrated brain cerebellar atrophy. Both clinical observations of 9400f thus agree with the diagnosis of autosomal recessive spastic ataxia of Charlevoix-Saguenay (Engert et al., 2000). Novel variants identified in this study enriched the repertoire of markers that are associated with different NDs.

Our TGP platform covers promoter regions that are often neglected in other panel-based or WES studies. Among all the known pathogenic variants in the ClinVar database, surprisingly, the c.-103C > T (ClinVar ID: 217166) non-coding variant in the 5′ UTR of *GJB1* was the most recurrent one in the cohort. Six patients from five families had this variant, which constitutes 4.4% of our patients. Five of those patients were males. Two of the patients were siblings (ID: 67067 and ID: 59e19), whereas the rest were unrelated. All of these except patient 5316c had a family history of CMT disease. The most commonly presented symptoms of these patients were weakness of distal limb muscles and decreased nerve conduction velocities, which indicate demyelination. In all cases, there were no atypical presentations of symptoms. The CMT neuropathy scores (CMTNS) (Murphy et al., 2011) were recorded from the patients. Three patients were classified as mild (CMTNS ≤ 10), and three patients were classified as moderate (CMTNS 11–20). Our study confirmed that the *GJB1* c.-103C > T variant in the promoter region was a major contributor in CMT disease in our cohort. Therefore, existing CMT diagnosis routines should be refined to include screening of the *GJB1* promoter.

### SCA35 Markers Showed Incomplete Penetrance and Imperfect Co-segregation

We observed two cases that contrasted with the existing knowledge of SCA35. Two pathogenic missense variants (D510H and L517W) in exon 10 of *TGM6* had previously been found to perfectly co-segregate with SCA35 symptoms (Wang et al., 2010; Li et al., 2013; Guo et al., 2014). The D510H variant was found in a 67-year-old Chinese female (ID: 7688b) who had been previously diagnosed with AD. The patient was also found to have the ApoE-ε4/ε4 haplotype, which confers a 25-fold higher risk of late-onset AD (Bertram et al., 2007). She presented with insidious onset of short-term memory decline, and her family has a history of late-onset dementia. Mild brain atrophy was observed on MRI scan, yet neither cerebellar dysfunction nor cerebellar atrophy was present. These negative findings suggested the incomplete penetrance of the D510H marker, and the major contributor to patient 7688b’s phenotype was the ApoE-ε4/ε4 haplotype.

For the L517W heterozygous variant, we observed two patients who demonstrated imperfect co-segregation. A 63-year-old female patient (ID: ef2d1) had a family history of cerebellar syndrome, and her MRI demonstrated mild cerebellar atrophy. However, further Sanger sequencing showed only one of her two affected relatives harbors the L517W variant, suggesting incomplete co-segregation with the phenotype. An unrelated 46-year-old male patient (ID: c2356) displayed gait disorder since age 28 with the clinical finding of spastic paraparesis. At age 37, progressive bulbar dysfunction appeared. Physical examination showed facial dystonia and blepharospasm. His MRI showed symmetrical bilateral T1 hypointense and T2 hyperintense signals at cerebellar hemispheres around the dentate nucleus with no contrast enhancement. Slightly prominent cerebellar folia, which may represent mild atrophic change, were also observed from MRI. Surprisingly, there was no history of cerebellar symptoms in the family of patient c2356.

Upon examining population genetics databases, the allele frequencies of D510H (1000G–0.3%; gnomAD–0.2%) and L517W (1000G–0.1%; gnomAD–0.2%) in East Asians were alarmingly high with regard to the incidence rate of SCA in general. As we were able to obtain three additional samples from the relatives of patient ef2d1, we first set out to further investigate if other variants could explain the phenotypes via WES. Family-based linkage analysis of patient ef2d1’s family (Tsoi et al., 2014) (Supplementary Figure S2) unexpectedly showed a negligible linkage disequilibrium (LD) signal at chromosome 20p13-12.2, which was reported as the LD block of SCA35 (Wang et al., 2010). We further searched for coding variants that fit the dominant mode of inheritance in the family within 20p13-12.2, yet all of them are common variants (minor allele frequency >1%) according to the 1000G and gnomAD databases. Based on the currently available results, we were unable to pinpoint the causative variant for this family. Our findings corroborate with a recent report of unaffected individuals who carry SCA35 markers, and a highly inflated prevalence of pathogenic variants in *TGM6* over the disease incidence rate of SCA (Fung et al., 2019). Further research may be required to elucidate if there are additional disease modifiers for SCA35.

### Comparison With Other Methods and Other Studies

Like NDs, the diagnosis of neurometabolic or neuromuscular syndromes is also impeded by non-specific clinical features. Using a panel of 614 genes that are related to familial neurometabolic syndromes, Reid et al. were able to identify the genetic diagnosis for 8 out of 21 (38%) undiagnosed patients, despite the absence of biochemical markers (Reid et al., 2016). In a cohort of 65 patients with spinal muscular atrophies but without mutation in *SMN1*, Karakaya et al. (2018) achieved 33% diagnostic yield with the help of a gene panel that contains 479 genes. These studies achieved a similar level of diagnostic yield when compared to our study.

Some commonly used sequencing panels for NDs cover a much smaller number of genes. For instance, Illumina’s TruSeq Neurodegeneration Panel covers 118 genes that are associated with seven groups of NDs. Cartagenia’s Treatable Metabolic Neurodegenerative Disorders panel covers 73 genes and 41 diseases. Meanwhile, ONDRISeq (Farhan et al., 2016) covers only 80 neuro-related genes.

WES and WGS cover a much larger portion of the genome. The broader scope of WES and WGS cause a higher level of uncertainty, insofar as many genomic regions have an unknown association with neurodegeneration and present difficulties associated with ascertaining the pathogenicity of a large amount of variants (Xue et al., 2015). Other trade-offs include the lower depth of coverage and higher cost (Xue et al., 2015; Reid et al., 2016).

WES and most existing neurodegeneration panels do not provide good coverage of CAG expansion loci. For instance, Nascimento et al. reported a case in which the WES result was normal, even though CAG expansion was present in *ATXN7* (Nascimento et al., 2018). Our panel provides coverage of known neurological repeats loci, which will increase the resolving power for diagnosis of NDs.

### Future Developments

Because of a high false positive rate of discovery, we did not cover CNVs and structural variations in this study. We envisage that further improvement in detection algorithms and sequencing technologies in the future could allow us to screen these variants as well. Theoretically, our STR expansion detection algorithm applies to all SCA types. Although we were able to benchmark the algorithm on patients with SCA3 only, the algorithm can be easily expanded to other STR expansion types once additional training data are available. With the ability to assess small variants and STR expansions in 311 genes simultaneously, our TGP platform holds tremendous potential to refine the diagnostic workflow of NDs.

## Data Availability Statement

The raw target-capture sequencing data were deposited to the European Genome-phenome Archive (EGA) under the accession EGAD00001005114. The access to the data is controlled due to privacy concerns. Interested parties can make a request to the corresponding author, HC, upon acceptance of the data access agreements on EGA. All variants reported in this article were submitted to ClinVar under the submission ID SUB5897755.

## Ethics Statement

The studies involving human participants were reviewed and approved by the Joint Chinese University of Hong Kong–New Territories East Cluster Clinical Research Ethics Committee (ref. no. CRE-2012.361). The patients/participants provided their written informed consent to participate in this study.

## Author Contributions

HC, T-FC, AC, and ACY designed the study. AC, LY, WA, TC, LC, and VM recruited the patients and performed genotyping. ACY, AKY, LY, TC, and J-WL performed the analysis, interpreted, and validated the results. ACY, AKY, AC, LY, WA, TC, XL, J-WL, HC, and T-FC prepared the manuscript. All authors contributed to the reviewing of the final version.

## Conflict of Interest

ACY and AKY are directors of Codex Genetics Limited. J-WL, T-FC, and HC are shareholders of Codex Genetics Limited. T-FC and HC receive compensation as scientific advisors of Codex Genetics Limited. The remaining authors declare that the research was conducted in the absence of any commercial or financial relationships that could be construed as a potential conflict of interest.
