# Supplementary material for: A Targeted Gene Panel That Covers Coding, Non-coding and Short Tandem Repeat Regions Improves the Diagnosis of Patients With Neurodegenerative Diseases
Source: Front Neurosci. 2019 Dec 11;13:1324. doi: 10.3389/fnins.2019.01324 (PMC6917647; doi:10.3389/fnins.2019.01324)
Supplement: Supplementary file 1 [file Table_1.DOCX]

Supplementary Material

# Supplementary Figures


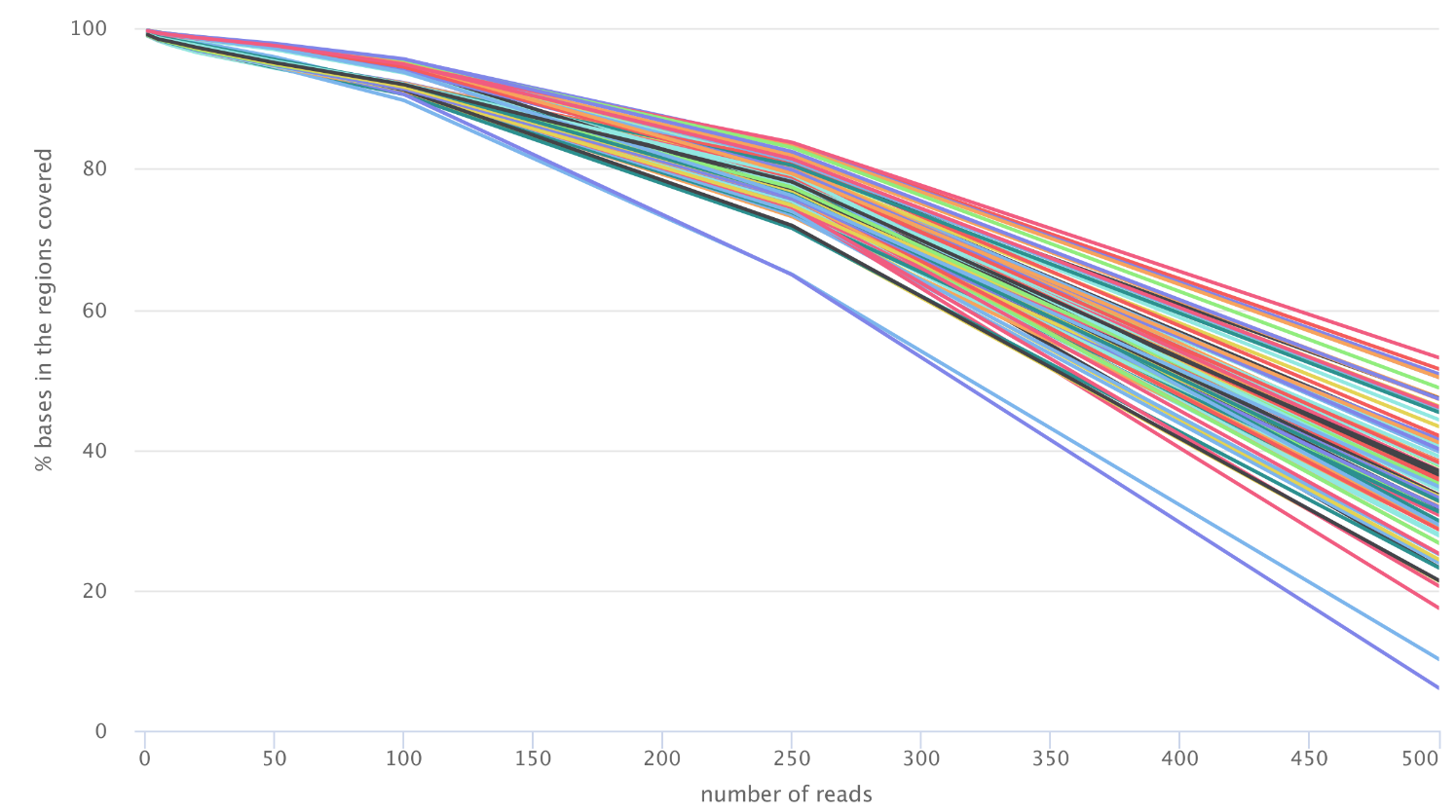


**Supplementary Figure 1.** Sequencing depth of coverage profile of the TGP.

**
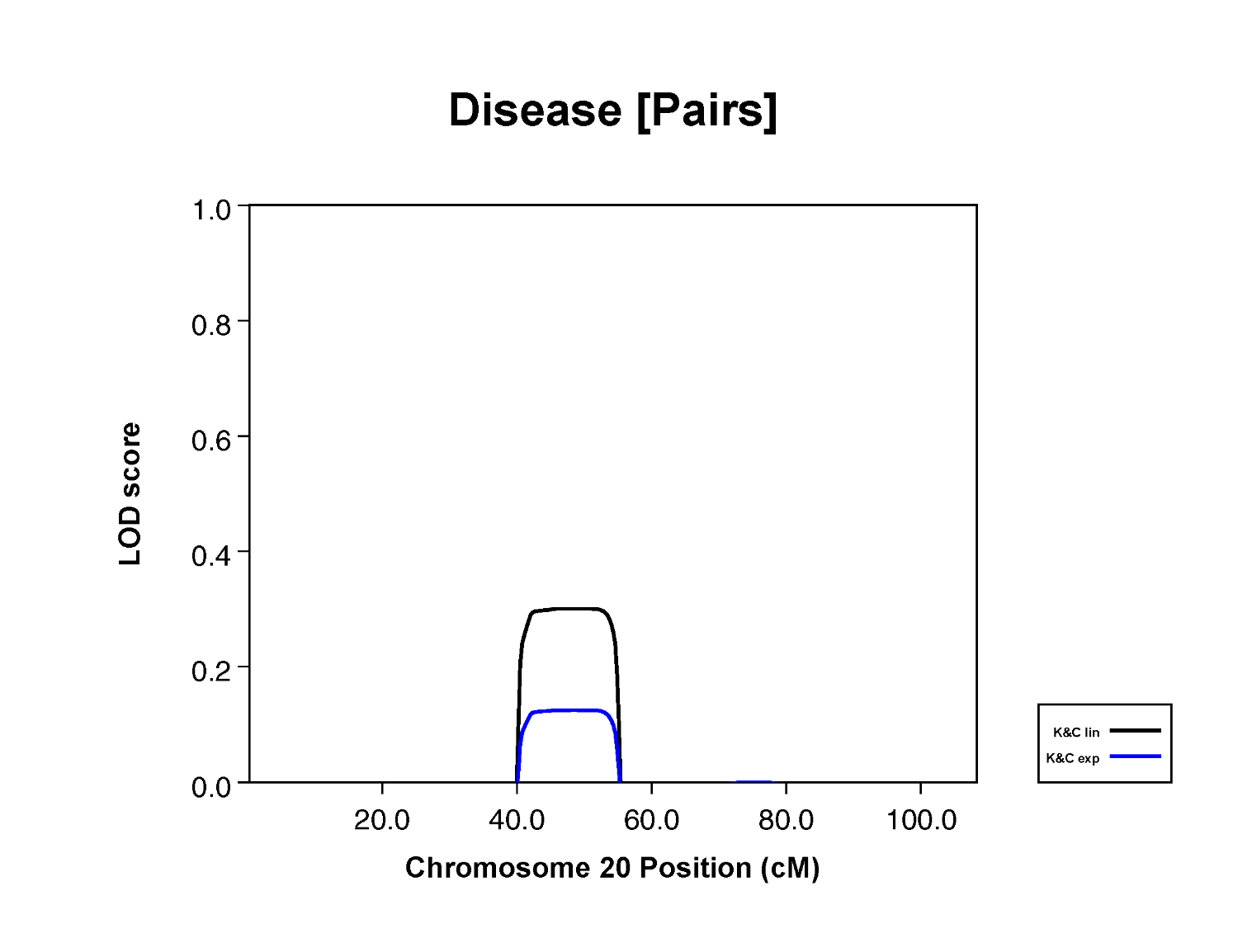
**

**Supplementary Figure 2.** MERLIN familial linkage analysis of ef2d1’s family. Only the logarithm of odds (LOD) score of chromosome 20, where the SCA35 marker resides, is plotted in this figure.

**
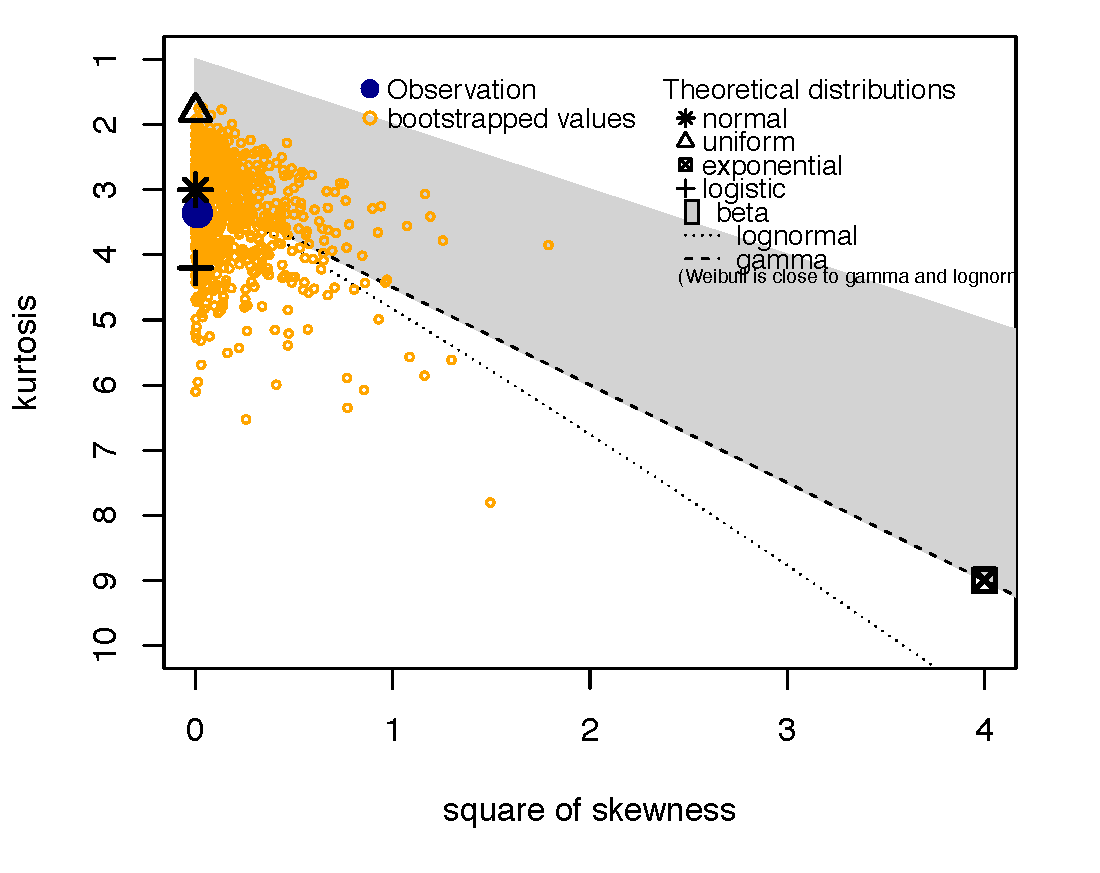
**

**Supplementary Figure 3.** Cullen and Frey graph of the normalized SCA3 read counts from the controls.

**
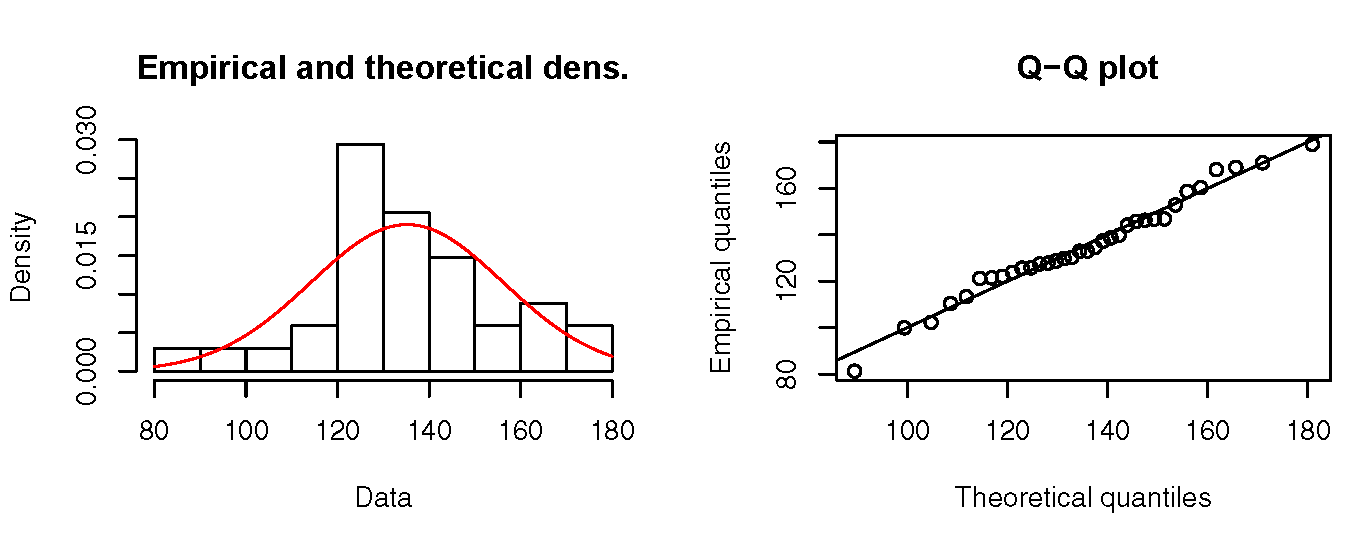
**

**Supplementary Figure 4.** Maximum likelihood fitting of the normalized SCA3 read counts from the controls to a normal distribution (left). Quantile-quantile (Q-Q) plot of the fitted distribution (right).

# Supplementary Tables

**Supplementary Table 1**. List of all subjects that were analysed using the TGP platform.

|  | **Patient ID** | **Ethnicity** | **Age** | **Preliminary diagnosis** | **Final diagnosis** | **Pathogenic/Likely Pathogenic variants** |
| --- | --- | --- | --- | --- | --- | --- |
| 1 | 4e074 | Asian | 61-65 | CEREBELLAR ATROPHY | SCA14 | Heterozygous PRKCG:c.301C>T:p.His101Tyr (rs121918511) |
| 2 | 4b227 | Asian | 51-55 | Familial cerebellar atrophy | N/A | N/A |
| 3 | ef2d1 | Asian | 61-65 | Spinocerebellar ataxia (unknown type) | SCA35 | Heterozygous TGM6:c.1550T>G:p.Leu517Trp (rs387907097) |
| 4 | e7f6c | Asian | 16-20 | Spinocerebellar ataxia (unknown type) | SCA11 | Heterozygous  TTBK2:c.1329dupA:p.Arg444ThrfsTer7 (rs80356538) |
| 5 | 79026 | Asian | 46-50 | Cerebellar degeneration | Cerebellar ataxia, neuropathy, epilepsy, and restless leg syndrome | Heterozygous POLG:c.2890C>T 5:p.Arg964Cys (rs201477273) |
| 6 | 2c624 | Chinese | 66-70 | PRESENILE DEMENTIA | N/A | N/A |
| 7 | 19581 | Chinese | 36-40 | SPASTIC PARAPARESIS | Cerebellar ataxia, neuropathy, epilepsy, and restless leg syndrome | Heterozygous POLG:c.2890C>T 5:p.Arg964Cys (rs201477273) |
| 8 | 4a44d | Chinese | 51-55 | SPASTIC PARAPARESIS ?HSP | N/A | N/A |
| 9 | 4fc82 | Chinese | 41-45 | PARKINSONISM | N/A | N/A |
| 10 | 6b51d | Chinese | 51-55 | PARKINSON'S DISEASE | N/A | N/A |
| 11 | 3fdba | Chinese | 41-45 | PARKINSON'S DISEASE | N/A | N/A |
| 12 | 8527a | Chinese | 66-70 | EARLY ONSET PARKINSON'S DISEASE | N/A | N/A |
| 13 | e629f | Chinese | 56-60 | SPASTIC PARAPARESIS ?HSP | N/A | N/A |
| 14 | b17ef | Chinese | 51-55 | CHARCOT-MARIE-TOOTH DISEASE | Charcot-Marie-Tooth Neuropathy X Type 1 | Hemizygous GJB1:c.-103C>T (rs863224971) |
| 15 | 45235 | Chinese | 16-20 | Hereditary spastic paraplegia | N/A | N/A |
| 16 | 4ec95 | Chinese | 51-55 | EARLY ONSET COGNITIVE DECLINE | N/A | N/A |
| 17 | 9400f | Chinese | 36-40 | ATAXIA AND SPASTIC GAIT | Spastic ataxia Charlevoix- Saguenay type / SPG33 | Compound heterozygous SACS:c.7504C>T:p.Arg2502* (rs281865118);  SACS:c.8132C>T:p.Ser2711Leu (Novel) |
| 18 | f5ca3 | Chinese | 41-45 | SPASTIC PARAPARESIS ?HSP | Alzheimer disease, familial, 3 | Heterozygous PSEN1:c.811C>G:Leu271Val (rs63750886) |
| 19 | 6f4b6 | Chinese | 56-60 | LIMB GIRDLE DYSTROPHY | N/A | N/A |
| 20 | 785f3 | Chinese | 61-65 | MOTOR NEURON DISEASE | ALS10 / Galactosylceramide beta-galactosidase deficiency | Heterozygous TARDBP:c.892G>A:p.Gly298Ser (rs4884357) Heterozygous GALC:c.1592G>A:p.Arg531His (rs200378205) |
| 21 | 535fa | Chinese | 56-60 | SPASTIC PARAPARESIS | N/A | N/A |
| 22 | c2356 | Chinese | 46-50 | DEMYELINATING DISEASE | SCA35 | Heterozygous TGM6:c.1550T>G:p.Leu517Trp (rs387907097) |
| 23 | b7a56 | Chinese | 61-65 | PARKINSON'S DISEASE | N/A | N/A |
| 24 | 5f9c4 | Chinese | 31-35 | SPASTIC PARAPARESIS | N/A | N/A |
| 25 | 67067 | Chinese | 51-55 | NEUROPATHY | Charcot-Marie-Tooth Neuropathy X Type 1 | Hemizygous GJB1:c.-103C>T (rs863224971) |
| 26 | 59e19 | Chinese | 41-45 | NEUROPATHY | Charcot-Marie-Tooth Neuropathy X Type 1 | Heterozygous GJB1:c.-103C>T (rs863224971) |
| 27 | 35135 | Chinese | 61-65 | DEMENTIA | N/A | N/A |
| 28 | 624b6 | Chinese | 26-30 | HEREDITARY SENSORY AND MOTOR NEUROPATHY TYPE II | N/A | N/A |
| 29 | eb1e3 | Chinese | 56-60 | FAMILIAL CEREBELLAR ATAXIA | N/A | N/A |
| 30 | e29c9 | Chinese | 51-55 | CHOREA, DEMENTIA | N/A | N/A |
| 31 | c6f3a | Chinese | 56-60 | CHARCOT-MARIE-TOOTH DISEASE | N/A | N/A |
| 32 | 86e50 | Chinese | 61-65 | CORPUS CALLOSUM ATROPHY, DEMENTIA | N/A | N/A |
| 33 | 9f140 | Chinese | 36-40 | SPASTIC PARAPARESIS | N/A | N/A |
| 34 | 76a50 | Chinese | 36-40 | ?SPINOCEREBELLAR ATAXIA | Parkinson disease 8, Cerebellar Ataxia | Heterozygous LRRK2:c.4883G>C: p.Arg1628Pro (rs33949390) Heterozygous POLG:c.2890C>T 5:p.Arg964Cys (rs201477273) |
| 35 | 7a61b | Chinese | 66-70 | SPASTIC PARAPARESIS | N/A | N/A |
| 36 | aea92 | Chinese | 51-55 | LEUCOENCEPHALOPATHY | N/A | N/A |
| 37 | 0b918 | Chinese | 56-60 | SPINOCEREBELLAR ATROPHY | N/A | N/A |
| 38 | d59ec | Chinese | 61-65 | MOTOR NEURON DISEASE | Parkinson disease 8, Klippel-Feil syndrome 1 | Heterozygous LRRK2:c.4883G>C: p.Arg1628Pro (rs33949390) Heterozygous GDF6:c.1271A>G: p.Lys424Arg (rs121909353) |
| 39 | 3d914 | Chinese | 56-60 | CEREBELLAR DEGENERATION | Spinocerebellar ataxia, autosomal recessive 8 | Compound heterozygous SYNE1:c.20263C>T:p.Arg6755Ter (rs780451185) SYNE1:c.8889delT:p.Gln2964AsnfsTer13 (Novel) |
| 40 | 73475 | Chinese | 51-55 | LEUCOENCEPHALOPATHY | Parkinson disease 8 | Heterozygous LRRK2:c.7153G>A: p.Gly2385Arg (rs34778348) |
| 41 | 44cb7 | Chinese | 46-50 | MULTIPLE SYSTEM ATROPHY | N/A | N/A |
| 42 | 71ee4 | Chinese | 66-70 | SPASTIC PARAPARESIS | Charcot-Marie-Tooth disease type 2F | Heterozygous HSPB1:c.250G>C:p.Gly84Arg (rs770272088) |
| 43 | 81178 | Chinese | 26-30 | CEREBELLAR DEGENERATION | N/A | N/A |
| 44 | 25fc0 | Chinese | 71-75 | BEHAVIORAL VARIANT FRONTOTEMPORAL DEMENTIA / MOTOR NEURON DISEASE | N/A | N/A |
| 45 | 31489 | Chinese | 66-70 | FRONTAL ALZHEIMER'S DISEASE | N/A | N/A |
| 46 | 98010 | Chinese | 61-65 | BEHAVIORAL VARIANT FRONTOTEMPORAL DEMENTIA | N/A | N/A |
| 47 | 0e17d | Chinese | 66-70 | ALZHEIMER'S DISEASE | N/A | N/A |
| 48 | 1a656 | Chinese | 71-75 | PROGRESSIVE NONFLUENT APHASIA | N/A | N/A |
| 49 | 031b4 | Chinese | 56-60 | ALZHEIMER'S DISEASE | Parkinson disease 8 | Heterozygous LRRK2:c.4883G>C: p.Arg1628Pro (rs33949390) |
| 50 | 41cfc | Chinese | 66-70 | SENILE DEMENTIA | N/A | N/A |
| 51 | 2858d | Chinese | 61-65 | SENILE DEMENTIA | N/A | N/A |
| 52 | 2fca3 | Chinese | 66-70 | MULTIPLE SYSTEM ATROPHY WITH CEREBELLAR FEATURES | N/A | N/A |
| 53 | 02d20 | Chinese | 61-65 | BEHAVIORAL VARIANT FRONTOTEMPORAL DEMENTIA | N/A | N/A |
| 54 | 7688b | Thai-Chinese | 66-70 | ALZHEIMER'S DISEASE | SCA35 / Alzheimer disease 2 | Heterozygous TGM6:c.1528G>C:p.Asp510His (rs201964784) Homozygous APOE:c.388T>C:p.Cys130Arg (rs429358) |
| 55 | c8376 | Chinese | 41-45 | BEHAVIORAL VARIANT FRONTOTEMPORAL DEMENTIA / PARKINSON'S DISEASE | Early-onset Alzheimer’s disease | Heterozygous PSEN1:c.781G>A:p.Val261Ile (Novel)  Heterozygous LRRK2:c.4883G>C: p.Arg1628Pro (rs33949390) |
| 56 | 6208e | Chinese | 61-65 | SENILE DEMENTIA | N/A | N/A |
| 57 | 3e1e9 | Chinese | 61-65 | FRONTOTEMPORAL DEMENTIA / PROGRESSIVE SUPRANUCLEAR PALSY | Parkinson disease 8 | Heterozygous LRRK2:c.4883G>C: p.Arg1628Pro (rs33949390) |
| 58 | 39fa9 | Chinese | 36-40 | NIEMANN PICK TYPE C1 | N/A | N/A |
| 59 | d029f | Chinese | 56-60 | VASCULAR DEMENTIA (LEFT FRONTAL INTRACRANIAL HEMORRHAGE) | N/A | N/A |
| 60 | 81b8a | Chinese | 61-65 | PROGRESSIVE NONFLUENT APHASIA / CORTICOBASAL DEGENERATION | N/A | N/A |
| 61 | da4ea | Chinese | 61-65 | BEHAVIORAL VARIANT FRONTOTEMPORAL DEMENTIA | N/A | N/A |
| 62 | a68b4 | Chinese | 51-55 | SENILE DEMENTIA | N/A | N/A |
| 63 | 108c9 | N/A |  |  | Parkinson disease 8 | Heterozygous LRRK2:c.4883G>C: p.Arg1628Pro (rs33949390) |
| 64 | 3ada9 | N/A |  |  | N/A | N/A |
| 65 | 49d18 | N/A |  |  | N/A | N/A |
| 66 | a2185 | N/A |  |  | N/A | N/A |
| 67 | c75cb | N/A |  |  | N/A | N/A |
| 68 | ff5a1 | N/A |  | Charcot-Marie-Tooth | N/A | N/A |
| 69 | 7f225 | N/A |  | Charcot-Marie-Tooth | Charcot-Marie-Tooth Neuropathy X Type 1 | Hemizygous GJB1:c.-103C>T (rs863224971) |
| 70 | 87226 | N/A |  | Frontotemporal Dementia | N/A | N/A |
| 71 | 96061 | N/A |  | Frontotemporal Dementia | N/A | N/A |
| 72 | eb624 | N/A |  | Frontotemporal Dementia | N/A | N/A |
| 73 | f369c | N/A |  | Frontotemporal Dementia | N/A | N/A |
| 74 | f74ef | N/A |  | Frontotemporal Dementia | N/A | N/A |
| 75 | a88a7 | N/A |  | Frontotemporal Dementia | N/A | N/A |
| 76 | 349c4 | Chinese | 56-70 | MITOCHONDRIAL DISEASE | N/A | N/A |
| 77 | 98a3a | Chinese | 41-45 | YOUNG ONSET PARKINSON'S DISEASE | N/A | N/A |
| 78 | 48449 | Chinese | 56-60 | CHARCOT MARIE TOOTH DISEASE TYPE 2 | N/A | N/A |
| 79 | 5316c | Chinese | 26-30 | CHARCOT MARIE TOOTH DISEASE TYPE 2 | Charcot-Marie-Tooth Neuropathy X Type 1 | Hemizygous GJB1:c.-103C>T (rs863224971) |
| 80 | a46e3 | Chinese | 21-25 | SMA | N/A | N/A |
| 81 | bbb96 | Chinese | 61-65 | YOUNG ONSET PARKINSON'S DISEASE | N/A | N/A |
| 82 | 44c80 | Chinese | 16-20 | CMT | Charcot-Marie-Tooth Neuropathy X Type 1 | Hemizygous GJB1:c.-103C>T (rs863224971) |
| 83 | b4944 | Chinese | 46-50 | MND FAMILY WITH ATAXIA | N/A | N/A |
| 84 | 434c9 | Chinese | 56-60 | MND FAMILY WITH ATAXIA | N/A | N/A |
| 85 | bdd2d | Chinese | 71-75 | CEREBELLAR ATROPHY | N/A | N/A |
| 86 | 8b940 | Chinese | 56-60 | PARKINSON'S DISESAE WITH FAMILY HISTORY | N/A | N/A |
| 87 | cd70b | Chinese | 51-55 | LEUCOENCEPHALOPATHY, SPASTIC ATAXIA | N/A | N/A |
| 88 | 69f59 | Chinese | 36-40 | YOUNG ONSET PARKINSON'S DISEASE | Parkinson disease 8, autosomal dominant | Heterozygous LRRK2:c.4883G>C: p.Arg1628Pro (rs33949390) |
| 89 | 1da51 | Chinese | 51-55 | HEREDITARY SPASTIC PARAPARESIS | Spastic paraplegia 4 autosomal dominant | Heterozygous CYP7B1:c.334C>T:p.Arg112Ter (rs200737038) Heterozygous SPAST:c.1507C>T:p.Arg503Trp (rs864622162) |
| 90 | 6e400 | Chinese | 56-60 | YOUNG ONSET PARKINSON'S DISEASE | N/A | N/A |
| 91 | e3d6c | Chinese | 51-55 | CEREBELLAR ATAXIA | Spinocerebellar ataxia 11 | Heterozygous TTBK2:c.1306_1307delGA:p.Asp436TyrfsTer14 (rs318240735) |
| 92 | ad48f | Chinese | 51-55 | MITROCHRONDIAL DISEASE | N/A | N/A |
| 93 | 7b1a2 | Chinese | 21-25 | PERIPHERAL NEUROPATHY, OPTIC ATROPHY | N/A | N/A |
| 94 | d6d82 | Chinese | 41-45 | HEREDITARY SPASTIC PARAPLEGIA | N/A | N/A |
| 95 | 29db0 | Chinese | 61-65 | CPEO ATAXIA PARKINSONISM | N/A | N/A |
| 96 | 82416 | Chinese | 66-70 | CHRONIC PROGRESSIVE EXTERNAL OPHTHALMOPLEGIA CPEO | N/A | N/A |
| 97 | 8c1f1 | Chinese | 46-50 | Charcot-Marie-Tooth disease (CMT) | N/A | N/A |
| 98 | ad573 | Chinese | 31-35 | Charcot-Marie-Tooth disease (CMT) | N/A | N/A |
| 99 | 16dc3 | Chinese | 36-40 | Charcot-Marie-Tooth disease (CMT) | N/A | N/A |
| 100 | 37834 | Chinese | 36-40 | Charcot-Marie-Tooth disease (CMT) | N/A | N/A |
| 101 | 454f6 | Chinese | 66-70 | EARLY ONSET ALZHEIMER'S DISEASE | N/A | N/A |
| 102 | 5ef6f | Chinese | 66-70 | EARLY ONSET MUSCULAR DYSTROPHY | N/A | N/A |
| 103 | 1253e | Chinese | 56-60 | ? MOTOR NEURON DISEASE | N/A | N/A |
| 104 | 482d9 | Chinese | 66-70 | MITROCHONDRIAL DISEASE | Renpenning syndrome 1 | Hemizygous PQBP1:c.461_462delAG:p.Glu154AlafsTer12 (rs606231195) |
| 105 | 3346f | Chinese | 56-60 | EARLY ONSET ALZHEIMER'S DISEASE | N/A | N/A |
| 106 | 9537f | Chinese | 51-55 | EARLY ALZHEIMER'S DISEASE WITH FAMILY HISTORY | N/A | N/A |
| 107 | 0fd42 | Chinese | 56-60 | CHARCOT–MARIE–TOOTH DISEASE | Charcot-Marie-Tooth Neuropathy X-linked;  Renpenning syndrome 1 | Hemizygous ENST00000361726.6:c.118G>T: p.Ala40Ser (Novel)  Hemizygous PQBP1:c.461_462delAG:p.Glu154AlafsTer12 (rs606231195) |
| 108 | 9bdb2 | Chinese | 61-65 | EARLY ONSET ALZHEIMER'S DISEASE | N/A | N/A |
| 109 | f6e0a | Chinese | 56-60 | EARLY ALZHEIMER'S DISEASE | N/A | N/A |
| 110 | b1556 | Chinese | 46-50 | Motor neuron disease | Amyotrophic lateral sclerosis type 10 | Heterozygous TARDBP:c.892G>A:p.Gly298Ser (rs4884357) |
| 111 | 6c658 | Chinese | 46-50 | EARLY ALZHEIMER'S DISEASE | N/A | N/A |
| 112 | 9f1f9 | Chinese | 51-55 | PRESENILE DEMENTIA | N/A | N/A |
| 113 | 28dae | Chinese | 66-70 | FAMILIAL ALZHEIMER DISEASE | N/A | N/A |
| 114 | e5b86 | Chinese | 51-55 | EARLY ALZHEIMER'S DISEASE | N/A | N/A |
| 115 | 2ac87 | Chinese | 66-70 | LEUCOENCEPHALOPATHY, AUTONOMAL DYSFUNCTION, VOMITING | N/A | N/A |
| 116 | CKK472-A | Unknown | 41-45 | Dystonia with parkinsonism features | N/A | N/A |
| 117 | 1735-A | Unknown | 36-40 |  | N/A | N/A |
| 118 | WYL451-B | Unknown | 71-75 |  | N/A | N/A |

**Supplementary Table 2**. List of genes covered by TGP and their associated diseases.

| **ICD-10 category ^1^** | **Gene** | **Description** | **Disease subtype** |
| --- | --- | --- | --- |
| Alzheimer Disease | APOE | apolipoprotein E | Alzheimer Disease Type 2 |
|  | APP | amyloid beta (A4) precursor protein | Alzheimer Disease Type 1 |
|  | PSEN1 | presenilin 1 | Alzheimer Disease Type 3 |
|  | PSEN2 | presenilin 2 (Alzheimer disease 4) | Alzheimer Disease Type 4 |
| Atypical virus infections of central nervous system | GSS | glutathione synthetase | Genetic Prion Diseases;Familial Creutzfeldt-Jakob Disease |
|  | PRNP | prion protein | Familial Creutzfeldt-Jakob Disease |
| Congenital malformations of spine and bony thorax | GDF6 | growth differentiation factor 6 | Klippel-Feil Syndrome |
| Disorders of aromatic amino-acid metabolism | FAH | fumarylacetoacetate hydrolase (fumarylacetoacetase) | Tyrosinemia Type I |
| Disorders of autonomic nervous system | IKBKAP | inhibitor of kappa light polypeptide gene enhancer in B-cells, kinase complex-associated protein | Familial Dysautonomia |
| Disorders of branched-chain amino-acid metabolism and fatty-acid metabolism | ABCD1 | ATP-binding cassette, sub-family D (ALD), member 1 | Adrenoleukodystrophy, X-Linked |
|  | AMN | amnionless homolog (mouse) | Adrenoleukodystrophy, X-Linked |
| Disorders of glycosaminoglycan metabolism | ARSB | arylsulfatase B | Mucopolysaccharidosis Type VI |
|  | EBP | emopamil binding protein (sterol isomerase) | Mucopolysaccharidosis Type IVB |
|  | GALNS | galactosamine (N-acetyl)-6-sulfate sulfatase | Mucopolysaccharidosis Type IVA |
|  | GAST | gastrin | Mucopolysaccharidosis Type IVA |
|  | GLB1 | galactosidase, beta 1 | Mucopolysaccharidosis Type IVB |
|  | GNS | glucosamine (N-acetyl)-6-sulfatase | Mucopolysaccharidosis Type IIID |
|  | GUSB | glucuronidase, beta | Mucopolysaccharidosis Type VII |
|  | HGSNAT | heparan-alpha-glucosaminide N-acetyltransferase | Mucopolysaccharidosis Type IIIC |
|  | HYAL1 | hyaluronoglucosaminidase 1 | Mucopolysaccharidosis Type IX |
|  | IDS | iduronate 2-sulfatase | Mucopolysaccharidosis Type II |
|  | IDUA | iduronidase, alpha-L- | Mucopolysaccharidosis Type I |
|  | NAGLU | N-acetylglucosaminidase, alpha | Mucopolysaccharidosis Type IIIB |
|  | NAT6 | N-acetyltransferase 6 (GCN5-related) | Mucopolysaccharidosis Type IX |
|  | SGSH | N-sulfoglucosamine sulfohydrolase | Mucopolysaccharidosis Type IIIA |
| Disorders of lipoprotein metabolism and other lipidaemias | MTTP | microsomal triglyceride transfer protein | Abetalipoproteinemia |
| Disorders of mineral metabolism | ATP7A | ATPase, Cu++ transporting, alpha polypeptide | Menkes Disease |
|  | ATP7B | ATPase, Cu++ transporting, beta polypeptide | Wilson Disease |
| Disorders of purine and pyrimidine metabolism | HPRT1 | hypoxanthine phosphoribosyltransferase 1 | Lesch-Nyhan Syndrome |
| Disorders of sphingolipid metabolism and other lipid storage disorders | ARSA | arylsulfatase A | Arylsulfatase A Deficiency |
|  | ASPA | aspartoacylase | Canavan Disease |
|  | CLN3 | ceroid-lipofuscinosis, neuronal 3 | Neuronal Ceroid-Lipofuscinoses |
|  | CLN5 | ceroid-lipofuscinosis, neuronal 5 | Neuronal Ceroid-Lipofuscinoses |
|  | CLN6 | ceroid-lipofuscinosis, neuronal 6, late infantile, variant | Neuronal Ceroid-Lipofuscinoses |
|  | CLN8 | ceroid-lipofuscinosis, neuronal 8 (epilepsy, progressive with mental retardation) | Neuronal Ceroid-Lipofuscinoses |
|  | CTSD | cathepsin D | Neuronal Ceroid-Lipofuscinoses |
|  | CYP27A1 | cytochrome P450, family 27, subfamily A, polypeptide 1 | cerebrotendinous xanthomatosis |
|  | DNAJC5 | DnaJ (Hsp40) homolog, subfamily C, member 5 | Neuronal Ceroid-Lipofuscinoses |
|  | GALC | galactosylceramidase | Krabbe Disease |
|  | GBA | glucosidase, beta, acid | Gaucher Disease |
|  | GFAP | glial fibrillary acidic protein | Alexander Disease |
|  | GLA | galactosidase, alpha | Fabry Disease |
|  | HEXB | hexosaminidase B (beta polypeptide) | Sandhoff Disease |
|  | LMNB1 | lamin B1 | Leukodystrophy, Adult-Onset, Autosomal Dominant |
|  | MFSD8 | major facilitator superfamily domain containing 8 | Neuronal Ceroid-Lipofuscinoses |
|  | NPC1 | Niemann-Pick disease, type C1 | Niemann-Pick Disease Type C |
|  | NPC2 | Niemann-Pick disease, type C2 | Niemann-Pick Disease Type C |
|  | PPT1 | palmitoyl-protein thioesterase 1 | Neuronal Ceroid-Lipofuscinosis |
|  | TPP1 | tripeptidyl peptidase I | Neuronal Ceroid-Lipofuscinosis |
| Dystonia | PRKRA | protein kinase, interferon-inducible double stranded RNA dependent activator | Dystonia 16 |
|  | RAX | retina and anterior neural fold homeobox | Dystonia 16 |
|  | SGCE | sarcoglycan, epsilon | Myoclonus-Dystonia |
|  | SLC2A1 | solute carrier family 2 (facilitated glucose transporter), member 1 | Dystonia 18 |
|  | THAP1 | THAP domain containing, apoptosis associated protein 1 | Dystonia 6 |
|  | TOR1A | torsin family 1, member A (torsin A) | Early-Onset Primary Dystonia |
| Epilepsy | ARX | aristaless related homeobox | X-Linked Infantile Spasm Syndrome |
|  | EPM2A | epilepsy, progressive myoclonus type 2A, Lafora disease (laforin) | Progressive Myoclonus Epilepsy, Lafora Type |
|  | FMN1 | formin 1 | Progressive Myoclonus Epilepsy, Lafora Type |
|  | NHLRC1 | NHL repeat containing 1 | Progressive Myoclonus Epilepsy, Lafora Type |
| Hereditary and idiopathic neuropathy | AARS | alanyl-tRNA synthetase | Charcot-Marie-Tooth Neuropathy Type 2 |
|  | CCT5 | chaperonin containing TCP1, subunit 5 (epsilon) | Autosomal Recessive Sensory Neuropathy with Spastic Paraplegia |
|  | DRG1 | developmentally regulated GTP binding protein 1 | Charcot-Marie-Tooth Neuropathy Type 4 |
|  | DYNC1H1 | dynein, cytoplasmic 1, heavy chain 1 | Charcot-Marie-Tooth Neuropathy Type 2 |
|  | EGR2 | early growth response 2 | Charcot-Marie-Tooth Neuropathy Type 1;Charcot-Marie-Tooth Neuropathy Type 4 |
|  | ERMAP | erythroblast membrane-associated protein (Scianna blood group) | Refsum Disease |
|  | FGD4 | FYVE, RhoGEF and PH domain containing 4 | Charcot-Marie-Tooth Neuropathy Type 4 |
|  | GARS | glycyl-tRNA synthetase | Charcot-Marie-Tooth Neuropathy Type 2 |
|  | GDAP1 | ganglioside induced differentiation associated protein 1 | Charcot-Marie-Tooth Neuropathy Type 2;Charcot-Marie-Tooth Neuropathy Type 4 |
|  | GJB1 | gap junction protein, beta 1, 32kDa | Charcot-Marie-Tooth Neuropathy X Type 1 |
|  | HSPB1 | heat shock 27kDa protein 1 | Charcot-Marie-Tooth Neuropathy Type 2 |
|  | HSPB8 | heat shock 22kDa protein 8 | Charcot-Marie-Tooth Neuropathy Type 2 |
|  | KIF1B | kinesin family member 1B | Charcot-Marie-Tooth Neuropathy Type 2 |
|  | LITAF | lipopolysaccharide-induced TNF factor | Charcot-Marie-Tooth Neuropathy Type 1 |
|  | LMNA | lamin A/C | Charcot-Marie-Tooth Neuropathy Type 2 |
|  | LRSAM1 | leucine rich repeat and sterile alpha motif containing 1 | Charcot-Marie-Tooth Neuropathy Type 2 |
|  | MED25 | mediator complex subunit 25 | Charcot-Marie-Tooth Neuropathy Type 2 |
|  | MFN2 | mitofusin 2 | Charcot-Marie-Tooth Neuropathy Type 2 |
|  | MPZ | myelin protein zero | Charcot-Marie-Tooth Neuropathy Type 1;Charcot-Marie-Tooth Neuropathy Type 2 |
|  | MTMR2 | myotubularin related protein 2 | Charcot-Marie-Tooth Neuropathy Type 4 |
|  | NDRG1 | N-myc downstream regulated 1 | Charcot-Marie-Tooth Neuropathy Type 4 |
|  | NEFL | neurofilament, light polypeptide | Charcot-Marie-Tooth Neuropathy Type 1 |
|  | PEX10 | peroxisomal biogenesis factor 10 | Refsum disease |
|  | PEX7 | peroxisomal biogenesis factor 7 | Refsum Disease |
|  | PHYH | phytanoyl-CoA 2-hydroxylase | Refsum Disease |
|  | PMP22 | peripheral myelin protein 22 | Charcot-Marie-Tooth Neuropathy Type 1 |
|  | PRPS1 | phosphoribosyl pyrophosphate synthetase 1 | Charcot-Marie-Tooth Neuropathy X |
|  | PRX | periaxin | Charcot-Marie-Tooth Neuropathy Type 4 |
|  | RAB7A | RAB7A, member RAS oncogene family | Charcot-Marie-Tooth Neuropathy Type 2 |
|  | SBF2 | SET binding factor 2 | Charcot-Marie-Tooth Neuropathy Type 4 |
|  | SH3TC2 | SH3 domain and tetratricopeptide repeats 2 | Charcot-Marie-Tooth Neuropathy Type 4 |
|  | SLC12A6 | solute carrier family 12 (potassium/chloride transporters), member 6 | Hereditary Motor and Sensory Neuropathy with Agenesis of the Corpus Callosum |
|  | SMAD1 | SMAD family member 1 | Charcot-Marie-Tooth Neuropathy Type 2 |
|  | SP110 | SP110 nuclear body protein | Charcot-Marie-Tooth Neuropathy Type 1 |
|  | TRPV4 | transient receptor potential cation channel, subfamily V, member 4 | Charcot-Marie-Tooth Neuropathy Type 2 |
| Hereditary ataxia | ADCK3 | aarF domain containing kinase 3 | Spinocerebellar Ataxia, Autosomal Recessive, 9 |
|  | AFG3L2 | AFG3 ATPase family gene 3-like 2 (S. cerevisiae) | Spinocerebellar Ataxia Type28 |
|  | APTX | aprataxin | Ataxia with Oculomotor Apraxia Type 1 |
|  | ATM | ataxia telangiectasia mutated | Ataxia Telangiectasia |
|  | ATN1 | atrophin 1 | Dentatorubral-pallidoluysian atrophy |
|  | ATP2B3 | ATPase, Ca++ transporting, plasma membrane 3 | Spinocerebellar Ataxia, X-Linked 1 |
|  | ATXN1 | ataxin 1 | Spinocerebellar Ataxia Type 1 |
|  | ATXN10 | ataxin 10 | Spinocerebellar Ataxia Type10 |
|  | ATXN2 | ataxin 2 | Spinocerebellar Ataxia Type 2 |
|  | ATXN3 | ataxin 3 | Spinocerebellar Ataxia Type 3 |
|  | ATXN7 | ataxin 7 | Spinocerebellar Ataxia Type 7 |
|  | ATXN8OS | ATXN8 opposite strand (non-protein coding) | Spinocerebellar Ataxia Type 8 |
|  | BEAN1 | brain expressed, associated with NEDD4, 1 | Spinocerebellar Ataxia Type 31 |
|  | C9orf72 | chromosome 9 open reading frame 72 | Corticobasal and ataxia syndromes |
|  | CACNA1A | calcium channel, voltage-dependent, P/Q type, alpha 1A subunit | Spinocerebellar Ataxia Type 6 |
|  | CCDC88C | coiled-coil domain containing 88C | Spinocerebellar Ataxia Type 40 |
|  | COX20 | COX20 cytochrome c oxidase assembly factor | Spinocerebellar Ataxia, Autosomal Recessive |
|  | CWF19L1 | CWF19-like 1, cell cycle control (S. pombe) | Spinocerebellar Ataxia, Autosomal Recessive 17 |
|  | DNAJC3 | DnaJ (Hsp40) homolog, subfamily C, member 3 | Ataxia, combined cerebellar and peripheral, with hearing loss and diabetes mellitus |
|  | EEF2 | eukaryotic translation elongation factor 2 | Spinocerebellar Ataxia Type 26 |
|  | ELOVL4 | ELOVL fatty acid elongase 4 | Spinocerebellar Ataxia Type 34 |
|  | ELOVL5 | ELOVL fatty acid elongase 5 | Spinocerebellar Ataxia Type 38 |
|  | FGF14 | fibroblast growth factor 14 | Spinocerebellar Ataxia Type27 |
|  | FXN | frataxin | Friedreich Ataxia |
|  | GRID2 | glutamate receptor, ionotropic, delta 2 | Spinocerebellar Ataxia, Autosomal Recessive 18 |
|  | HPCA | hippocalcin | Spinocerebellar Ataxia Type 6 |
|  | IFRD1 | interferon-related developmental regulator 1 | Spinocerebellar Ataxia Type18 |
|  | ITPR1 | inositol 1,4,5-trisphosphate receptor, type 1 | Spinocerebellar Ataxia Type15 |
|  | KCNA1 | potassium channel, voltage gated shaker related subfamily A, member 1 | episodic ataxia type 1 |
|  | KCNC3 | potassium voltage-gated channel, Shaw-related subfamily, member 3 | Spinocerebellar Ataxia Type13 |
|  | KCND3 | potassium channel, voltage gated Shal related subfamily D, member 3 | Spinocerebellar Ataxia Type 19,22 |
|  | KCNN3 | potassium channel, two pore domain subfamily K, member 13 | Sporadic Ataxia |
|  | NBN | nibrin | Nijmegen Breakage Syndrome |
|  | NOP56 | NOP56 ribonucleoprotein | Spinocerebellar Ataxia Type 36 |
|  | PDYN | prodynorphin | Spinocerebellar Ataxia Type23 |
|  | PLEKHG4 | pleckstrin homology domain containing, family G (with RhoGef domain) member 4 | Spinocerebellar Ataxia Type 4 |
|  | PPP2R2B | protein phosphatase 2, regulatory subunit B, beta | Spinocerebellar Ataxia Type12 |
|  | PRKCG | protein kinase C, gamma | Spinocerebellar Ataxia Type14 |
|  | RNF216 | ring finger protein 216 | Ataxia |
|  | SACS | sacsin molecular chaperone | Autosomal recessive spastic ataxia of Charlevoix-Saguenay |
|  | SLC1A3 | solute carrier family 1 (glial high affinity glutamate transporter), member 3 | episodic ataxia |
|  | SLC1A6 | solute carrier family 1 (high affinity aspartate/glutamate transporter), member 6 | Spinocerebellar Ataxia Type 5,23 |
|  | SNAP25 | synaptosomal-associated protein, 25kDa | Sporadic Ataxia |
|  | SNX14 | sorting nexin 14 | Hereditary cerebellar ataxias, Autosomal recessive |
|  | SPG20 | spastic paraplegia 20 (Troyer syndrome) | Troyer Syndrome |
|  | SPTBN2 | spectrin, beta, non-erythrocytic 2 | Spinocerebellar Ataxia Type 5 |
|  | STUB1 | STIP1 homology and U-box containing protein 1, E3 ubiquitin protein ligase | Spinocerebellar Ataxia, Autosomal Recessive 16 |
|  | SYNE1 | spectrin repeat containing, nuclear envelope 1 | SYNE1-Related Autosomal Recessive Cerebellar Ataxia |
|  | TBP | TATA box binding protein | Spinocerebellar Ataxia Type17 |
|  | TDP1 | tyrosyl-DNA phosphodiesterase 1 | Spinocerebellar Ataxia with Axonal Neuropathy, Autosomal Recessive 23 |
|  | TGM6 | transglutaminase 6 | Spinocerebellar Ataxia Type 35 |
|  | TMEM240 | transmembrane protein 240 | Spinocerebellar Ataxia Type 21 |
|  | TRPC3 | Transient Receptor Potential Cation Channel, Subfamily C, Member 3 | Spinocerebellar Ataxia Type 41 |
|  | TTBK2 | tau tubulin kinase 2 | Spinocerebellar Ataxia Type11 |
|  | TTPA | tocopherol (alpha) transfer protein | Ataxia with Vitamin E Deficiency |
|  | UBR4 | ubiquitin protein ligase E3 component n-recognin 4 | episodic ataxia type 8 |
|  | UEVLD | UEV and lactate/malate dehyrogenase domains | Ataxia with Vitamin E Deficiency |
|  | VAMP1 | vesicle-associated membrane protein 1 (synaptobrevin 1) | Spastic ataxia-1 |
|  | WWOX | WW domain containing oxidoreductase | Spinocerebellar Ataxia, Autosomal Recessive 12 |
| Huntington Disease | HTT | huntingtin | Huntington Disease |
|  | JPH3 | Junctophilin 3 | Huntington Disease Like 2 |
|  | SLC6A4 | solute carrier family 6 (neurotransmitter transporter, serotonin), member 4 | Huntington Disease |
| Microcephaly | ASPM | asp (abnormal spindle) homolog, microcephaly associated (Drosophila) | Primary Autosomal Recessive Microcephaly |
|  | CDK5RAP2 | CDK5 regulatory subunit associated protein 2 | Primary Autosomal Recessive Microcephaly Type 3 |
|  | CENPJ | centromere protein J | Primary Autosomal Recessive Microcephaly Type 6 |
|  | CEP152 | centrosomal protein 152kDa | Primary Autosomal Recessive Microcephaly Type 4 |
|  | MCPH1 | microcephalin 1 | Primary Autosomal Recessive Microcephaly |
|  | PMEL | premelanosome protein | Primary Autosomal Recessive Microcephaly |
|  | STIL | SCL/TAL1 interrupting locus | Primary Autosomal Recessive Microcephaly Type 7 |
|  | WDR62 | WD repeat domain 62 | Primary Autosomal Recessive Microcephaly Type 2 |
| Monosomies and deletions from the autosomes, not elsewhere classified | HLA-B | major histocompatibility complex, class I, B | Angelman Syndrome |
|  | UBE3A | ubiquitin protein ligase E3A | Angelman Syndrome |
| Nerve root and plexus disorders | SEPT9 | septin 9 | Hereditary Neuralgic Amyotrophy |
| Other anaemias | ABCB7 | ATP-binding cassette, sub-family B (MDR/TAP), member 7 | X-Linked Sideroblastic Anemia and Ataxia |
| Other chromosome abnormalities, not elsewhere classified | FMR1 | fragile X mental retardation 1 | Fragile X-associated tremor/ataxia syndrome |
| Other congenital malformations of brain | AHI1 | Abelson helper integration site 1 | Joubert Syndrome |
|  | ARL13B | ADP-ribosylation factor-like 13B | Joubert Syndrome |
|  | CC2D2A | coiled-coil and C2 domain containing 2A | Joubert Syndrome |
|  | CEP290 | centrosomal protein 290kDa | Joubert Syndrome |
|  | EMX2 | empty spiracles homeobox 2 | Familial Schizencephaly |
|  | FOXH1 | forkhead box H1 | Holoprosencephaly |
|  | GLI2 | GLI family zinc finger 2 | Holoprosencephaly |
|  | HESX1 | HESX homeobox 1 | Septooptic Dysplasia |
|  | INPP5E | inositol polyphosphate-5-phosphatase, 72 kDa | Joubert Syndrome |
|  | KIF7 | kinesin family member 7 | Joubert Syndrome |
|  | NDE1 | nudE nuclear distribution E homolog 1 (A. nidulans) | Lissencephaly 4 |
|  | NODAL | nodal homolog (mouse) | Holoprosencephaly |
|  | NPHP1 | nephronophthisis 1 (juvenile) | Joubert Syndrome |
|  | OFD1 | oral-facial-digital syndrome 1 | Joubert Syndrome |
|  | PMPCA | peptidase (mitochondrial processing) alpha | Joubert Syndrome |
|  | PTCH1 | patched 1 | Holoprosencephaly |
|  | RELN | reelin | Lissencephaly 2 |
|  | RPGRIP1L | RPGRIP1-like | Joubert Syndrome |
|  | SHH | sonic hedgehog | Holoprosencephaly |
|  | SIX3 | SIX homeobox 3 | Holoprosencephaly |
|  | TCTN1 | tectonic family member 1 | Joubert Syndrome |
|  | TCTN2 | tectonic family member 2 | Joubert Syndrome |
|  | TGIF1 | TGFB-induced factor homeobox 1 | Holoprosencephaly |
|  | TMEM216 | transmembrane protein 216 | Joubert Syndrome |
|  | TMEM67 | transmembrane protein 67 | Joubert Syndrome |
|  | TUBA1A | tubulin, alpha 1a | Lissencephaly 3 |
|  | ZIC2 | Zic family member 2 | Holoprosencephaly |
| Other congenital malformations of skin | IKBKG | inhibitor of kappa light polypeptide gene enhancer in B-cells, kinase gamma | Incontinentia Pigmenti |
| Other congenital malformations of skull and face bones | FGFR1 | fibroblast growth factor receptor 1 | FGFR-Related Craniosynostosis |
|  | FGFR2 | fibroblast growth factor receptor 2 | FGFR-Related Craniosynostosis |
|  | FGFR3 | fibroblast growth factor receptor 3 | FGFR-Related Craniosynostosis |
|  | MSX2 | msh homeobox 2 | Craniosynostosis, Type 2 |
|  | TWIST1 | twist homolog 1 (Drosophila) | Craniosynostosis, Type 1 |
| Other degenerative diseases of nervous system, not elsewhere classified | BCS1L | BCS1-like (S. cerevisiae) | Leigh Syndrome |
|  | COX10 | COX10 homolog, cytochrome c oxidase assembly protein, heme A: farnesyltransferase (yeast) | Leigh Syndrome |
|  | DLD | dihydrolipoamide dehydrogenase | Leigh Syndrome |
|  | NDUFA1 | NADH dehydrogenase (ubiquinone) 1 alpha subcomplex, 1, 7.5kDa | Leigh Syndrome |
|  | NDUFAF2 | NADH dehydrogenase (ubiquinone) 1 alpha subcomplex, assembly factor 2 | Leigh Syndrome |
|  | NDUFS1 | NADH dehydrogenase (ubiquinone) Fe-S protein 1, 75kDa (NADH-coenzyme Q reductase) | Leigh Syndrome |
|  | NDUFS4 | NADH dehydrogenase (ubiquinone) Fe-S protein 4, 18kDa (NADH-coenzyme Q reductase) | Leigh Syndrome |
|  | NDUFS7 | NADH dehydrogenase (ubiquinone) Fe-S protein 7, 20kDa (NADH-coenzyme Q reductase) | Leigh Syndrome |
|  | NDUFS8 | NADH dehydrogenase (ubiquinone) Fe-S protein 8, 23kDa (NADH-coenzyme Q reductase) | Leigh Syndrome |
|  | NDUFV1 | NADH dehydrogenase (ubiquinone) flavoprotein 1, 51kDa | Leigh Syndrome |
|  | PDHA1 | pyruvate dehydrogenase (lipoamide) alpha 1 | X-Linked Leigh Syndrome |
|  | POLG | polymerase (DNA directed), gamma | Alpers-Huttenlocher Syndrome |
|  | SCO1 | SCO cytochrome oxidase deficient homolog 1 (yeast) | Leigh Syndrome |
|  | SCO2 | SCO cytochrome oxidase deficient homolog 2 (yeast) | Leigh Syndrome |
|  | SDHA | succinate dehydrogenase complex, subunit A, flavoprotein (Fp) | Leigh Syndrome |
|  | SURF1 | surfeit 1 | Leigh Syndrome |
| Other disorders of brain | CDKL5 | cyclin-dependent kinase-like 5 | Epileptic Encephalopathy, Early Infantile, 2 |
|  | ETHE1 | ethylmalonic encephalopathy 1 | Ethylmalonic Encephalopathy |
|  | STXBP1 | syntaxin binding protein 1 | Epileptic Encephalopathy, Early Infantile, 4 |
| Other extrapyramidal and movement disorders | NOL3 | nucleolar protein 3 (apoptosis repressor with CARD domain) | Familial cortical myoclonus |
| Other myopathies | GNE | glucosamine (UDP-N-acetyl)-2-epimerase/N-acetylmannosamine kinase | Inclusion Body Myopathy 2 |
|  | MYH2 | myosin, heavy chain 2, skeletal muscle, adult | Inclusion Body Myopathy 3 |
| Other osteochondrodysplasias | TGFB1 | transforming growth factor, beta 1 | Camurati-Engelmann Disease |
| Other specified congenital malformation syndromes affecting multiple systems | NSD1 | nuclear receptor binding SET domain protein 1 | Sotos Syndrome |
| Others | CHRDL2 | chordin-like 2 |  |
|  | ELN | elastin | Williams Syndrome |
|  | GPR56 | G protein-coupled receptor 56 | Polymicrogyria |
|  | GRIN2A | glutamate receptor, ionotropic, N-methyl D-aspartate 2A |  |
|  | HSD17B4 | hydroxysteroid (17-beta) dehydrogenase 4 | HSD17B4-deficiency |
|  | HYLS1 | hydrolethalus syndrome 1 | Hydrolethalus Syndrome 1 |
|  | KCNK13 | potassium channel, calcium activated intermediate/small conductance subfamily N alpha, member 3 |  |
|  | NAGA | N-acetylgalactosaminidase, alpha- | Schindler Disease |
|  | PLA2G6 | phospholipase A2, group VI (cytosolic, calcium-independent) |  |
|  | PSAP | prosaposin | Saposin B Deficiency |
|  | SLC9A1 | solute carrier family 9, subfamily A (NHE1, cation proton antiporter 1), member 1 | Lichtenstein-Knorr syndrome |
|  | TIMM8A | translocase of inner mitochondrial membrane 8 homolog A (yeast) | Deafness-Dystonia-Optic Neuronopathy Syndrome |
|  | VANGL1 | vang-like 1 (van gogh, Drosophila) | VANGL1-Related Neural Tube Defect |
| Paraplegia and tetraplegia | ATL1 | atlastin GTPase 1 | Spastic Paraplegia 3A |
|  | B4GALNT1 | beta-1,4-N-acetyl-galactosaminyl transferase 1 | spastic paraplegia-26 |
|  | CYP2U1 | cytochrome P450, family 2, subfamily U, polypeptide 1 | spastic paraplegia-56 |
|  | CYP7B1 | cytochrome P450, family 7, subfamily B, polypeptide 1 | Spastic Paraplegia 5A |
|  | DDHD1 | DDHD domain containing 1 | spastic paraplegia-28 |
|  | DDHD2 | DDHD domain containing 2 | spastic paraplegia-28 |
|  | FA2H | fatty acid 2-hydroxylase | spastic paraplegia-35 |
|  | GBA2 | glucosidase, beta (bile acid) 2 | spastic paraplegia-46 |
|  | GJC2 | gap junction protein, gamma 2, 47kDa | Spastic Paraplegia 44 |
|  | HSPD1 | heat shock 60kDa protein 1 (chaperonin) | Spastic Paraplegia 13 |
|  | KIAA0196 | KIAA0196 | Spastic Paraplegia 8 |
|  | KIF5A | kinesin family member 5A | Spastic Paraplegia 10 |
|  | NIPA1 | non imprinted in Prader-Willi/Angelman syndrome 1 | Spastic Paraplegia 6 |
|  | PNPLA6 | patatin-like phospholipase domain containing 6 | Spastic Paraplegia 39 |
|  | REEP1 | receptor accessory protein 1 | Spastic Paraplegia 31 |
|  | SLC33A1 | solute carrier family 33 (acetyl-CoA transporter), member 1 | Spastic Paraplegia 42 |
|  | SPAST | spastin | Spastic Paraplegia 4 |
|  | SPG11 | spastic paraplegia 11 (autosomal recessive) | Spastic Paraplegia 11 |
|  | SPG21 | spastic paraplegia 21 (autosomal recessive, Mast syndrome) | Mast Syndrome |
|  | SPG7 | spastic paraplegia 7 (pure and complicated autosomal recessive) | Spastic Paraplegia 7 |
|  | ZFYVE26 | zinc finger, FYVE domain containing 26 | Spastic Paraplegia 15 |
|  | ZFYVE27 | zinc finger, FYVE domain containing 27 | Spastic Paraplegia 33 |
| Parkinson disease | ATP13A2 | ATPase type 13A2 | Kufor-Rakeb Syndrome |
|  | HTRA2 | HtrA serine peptidase 2 | Parkinson Disease |
|  | LRRK2 | leucine-rich repeat kinase 2 | Parkinson Disease |
|  | NR4A2 | nuclear receptor subfamily 4, group A, member 2 | Parkinson Disease |
|  | PARK2 | parkinson protein 2, E3 ubiquitin protein ligase (parkin) | Parkinson Disease |
|  | PARK7 | parkinson protein 7 | Parkinson Disease |
|  | PINK1 | PTEN induced putative kinase 1 | Parkinson Disease |
|  | SNCA | synuclein, alpha (non A4 component of amyloid precursor) | Parkinson Disease |
|  | SNCAIP | synuclein, alpha interacting protein | Parkinson Disease |
|  | UCHL1 | ubiquitin carboxyl-terminal esterase L1 (ubiquitin thiolesterase) | Parkinson Disease |
| Pervasive developmental disorders | NLGN3 | neuroligin 3 | Autistic Disorder |
|  | NLGN4X | neuroligin 4, X-linked | Autistic Disorder |
|  | SNRPN | small nuclear ribonucleoprotein polypeptide N | Autistic Disorder |
| Phakomatoses, not elsewhere classified | NF1 | neurofibromin 1 | Neurofibromatosis-Noonan Syndrome |
|  | NF2 | neurofibromin 2 (merlin) | Neurofibromatosis 2 |
|  | SPRED1 | sprouty-related, EVH1 domain containing 1 | Legius Syndrome |
|  | TSC1 | tuberous sclerosis 1 | Tuberous Sclerosis 1 |
|  | TSC2 | tuberous sclerosis 2 | Tuberous Sclerosis 2 |
| Primary disorders of muscles | BIN1 | bridging integrator 1 | Centronuclear Myopathy 2 |
|  | CLCN1 | chloride channel, voltage-sensitive 1 | Myotonia Congenita |
|  | CNBP | CCHC-Type Zinc Finger, Nucleic Acid Binding Protein | myotonic dystrophy type 2 |
|  | DMPK | dystrophia myotonica protein kinase | Myotonic Dystrophy Type 1 |
|  | DNM2 | dynamin 2 | Centronuclear Myopathy 1 |
|  | MTM1 | myotubularin 1 | X-Linked Centronuclear Myopathy |
| Schizophrenia | FXYD6 | FXYD domain containing ion transport regulator 6 | Schizophrenia |
|  | PRODH | proline dehydrogenase (oxidase) 1 | Schizophrenia |
|  | PRODH2 | proline dehydrogenase (oxidase) 2 | Schizophrenia |
|  | TAAR6 | trace amine associated receptor 6 | Schizophrenia |
| Secondary parkinsonism | ATP1A3 | ATPase, Na+/K+ transporting, alpha 3 polypeptide | Rapid-Onset Dystonia-Parkinsonism |
| Specific developmental disorders of speech and language | FOXP2 | forkhead box P2 | Speech-Language Disorder 1 |
| Spinal muscular atrophy and related syndromes | ALS2 | amyotrophic lateral sclerosis 2 (juvenile) | Amyotrophic Lateral Sclerosis |
|  | ANG | angiogenin, ribonuclease, RNase A family, 5 | Amyotrophic Lateral Sclerosis |
|  | AR | androgen receptor | Spinal and Bulbar Muscular Atrophy |
|  | FIG4 | FIG4 homolog, SAC1 lipid phosphatase domain containing (S. cerevisiae) | Amyotrophic Lateral Sclerosis |
|  | FUS | fused in sarcoma | Amyotrophic Lateral Sclerosis |
|  | OPTN | optineurin | Amyotrophic Lateral Sclerosis |
|  | SETX | senataxin | Amyotrophic Lateral Sclerosis |
|  | SMN1 | survival of motor neuron 1, telomeric | Spinal Muscular Atrophy |
|  | SMN2 | survival of motor neuron 2, centromeric | Spinal Muscular Atrophy |
|  | SOD1 | superoxide dismutase 1, soluble | Amyotrophic Lateral Sclerosis |
|  | TARDBP | TAR DNA binding protein | Amyotrophic Lateral Sclerosis |
|  | VAPB | VAMP (vesicle-associated membrane protein)-associated protein B and C | Amyotrophic Lateral Sclerosis |
|  | VCP | valosin containing protein | Amyotrophic Lateral Sclerosis |
| Tic disorders | SLITRK1 | SLIT and NTRK-like family, member 1 | Tourette Syndrome |
| Unspecified dementia | DNMT1 | DNA (cytosine-5-)-methyltransferase 1 | Dementia |
|  | ITM2B | integral membrane protein 2B | familial dementia |
| Unspecified mental retardation | ATP6AP2 | ATPase, H+ transporting, lysosomal accessory protein 2 | X-Linked Mental Retardation with Epilepsy |
|  | PQBP1 | polyglutamine binding protein 1 | Renpenning Syndrome 1 |
|  | RPS6KA3 | ribosomal protein S6 kinase, 90kDa, polypeptide 3 | Coffin-Lowry Syndrome |
|  | SLC9A6 | solute carrier family 9, subfamily A (NHE6, cation proton antiporter 6), member 6 | X-linked mental retardation |

Note: ^1^ ICD-10, International Statistical Classification of Diseases and Related Health Problems 10th Revision (World Health Organization, 2016)
